# Characterization of a non-nudix pyrophosphatase points to interplay between flavin and NAD(H) homeostasis in *Saccharomyces cerevisiae*

**DOI:** 10.1371/journal.pone.0198787

**Published:** 2018-06-14

**Authors:** Joseph H. Lynch, Na Sa, Sompop Saeheng, Nadia Raffaelli, Sanja Roje

**Affiliations:** 1 Institute of Biological Chemistry, Washington State University, Pullman, WA, United States of America; 2 Dipartimento di Scienze Agrarie, Alimentari e Ambientali, Università Politecnica delle Marche, Ancona, Italy; Johns Hopkins University School of Medicine, UNITED STATES

## Abstract

The flavin cofactors FMN and FAD are required for a wide variety of biological processes, however, little is known about their metabolism. Here, we report the cloning and biochemical characterization of the *Saccharomyces cerevisiae* pyrophosphatase Fpy1p. Genetic and functional studies suggest that Fpy1p may play a key role in flavin metabolism and is the first-reported non-Nudix superfamily enzyme to display FAD pyrophosphatase activity. Characterization of mutant yeast strains found that deletion of *fpy1* counteracts the adverse effects that are caused by deletion of *flx1*, a known mitochondrial FAD transporter. We show that Fpy1p is capable of hydrolyzing FAD, NAD(H), and ADP-ribose. The enzymatic activity of Fpy1p is dependent upon the presence of K^+^ and divalent metal cations, with similar kinetic parameters to those that have been reported for Nudix FAD pyrophosphatases. In addition, we report that the deletion of *fpy1* intensifies the FMN-dependence of null mutants of the riboflavin kinase Fmn1p, demonstrate that *fpy1* mutation abolishes the decreased fitness resulting from the deletion of the *flx1* ORF, and offer a possible mechanism for the genetic interplay between *fpy1*, *flx1* and *fmn1*.

## Introduction

The riboflavin derivatives flavin adenine dinucleotide (FAD) and flavin mononucleotide (FMN) are critical enzyme cofactors in all living organisms. FAD and FMN are required for a wide variety of metabolic processes, some of which include mitochondrial electron transport, antioxidant reduction, protein folding, chromatin remodeling, as well as the metabolism of nucleotides, amino acids, other cofactors, and numerous other biologically important compounds [1–4]. Plants further rely on these cofactors for specialized functions such as blue-light signaling and photosynthesis [4–7].

Riboflavin is synthesized from GTP and ribulose-5-phosphate. This biosynthetic pathway is absent in humans and other *Animalia*, but has been characterized in yeast, bacteria and plants [4,8–12]. FMN is then synthesized from riboflavin via ATP-dependent phosphorylation by riboflavin kinases; FAD is subsequently formed from FMN through ATP-dependent adenylylation by FAD synthetases [9,10,13–16]. The interconversion of flavin nucleotides is further mediated by FAD pyrophosphatases, which cleave AMP from FAD to re-form FMN, as well as FMN hydrolases, which hydrolyze inorganic phosphate from FMN to yield the original riboflavin precursor [14,15,17–22].

Enzymes that interconvert riboflavin, FMN, and FAD may critically affect the balance and availability of flavin cofactors throughout the cell. Despite this importance, little is known about these enzymes, their interplay with other metabolic processes, or their true cellular location. Previous studies found that eukaryotic mitochondria and cytosol each possess a distinct set of flavin-interconverting-enzymes, however, many of the catalysts whose activity is described in these studies have yet to be identified [15,19,23–26].

Yeast are an excellent model organism for the study of riboflavin metabolism. In *Saccharomyces cerevisiae*, FMN is synthesized by a single-known riboflavin kinase, Fmn1p [27]. Results of Fmn1p–GFP fusion studies indicate that Fmn1p only resides in the mitochondria; however, immunolocalization studies suggest Fmn1p may be located both in the mitochondria and microsomes, the latter of which has been attributed to the presence of Fmn1p on the cytoplasmic face of the endoplasmic reticulum membrane [27]. *S*. *cerevisiae* also contain only a single-known FAD synthetase, Fad1p [28]. In contrast to Fmn1p, Fad1p–GFP fusion studies suggest that the enzyme is normally localized to the cytosol [29]. Fad1p has been observed in the mitochondria of yeast that overexpress the enzyme, however the authors attributed this finding to mislocalization of the protein, likely caused by overabundance [28].

While many organisms, including yeast, are capable of hydrolyzing FMN and FAD, few of the corresponding genes have been identified for the enzymes that underlie this activity [11,17–19,26,30–36]. All of the known enzymes with FAD pyrophosphatase activity belong to the Nudix superfamily, which has wide phylogenetic distribution [31–33,36,37]. Members of this superfamily act on a broad array of nucleotide disphosphate motifs, and although several hydrolyze FAD, none are completely FAD-specific [38]. Despite the likelihood that non-Nudix nucleotide pyrophosphatases exist that are capable of hydrolyzing FAD, the current evidence is limited to a single report of an enzyme in bacteria for which FAD is not the preferred substrate [39].

In addition to interest in the identification/characterization of novel flavin-interconverting-enzymes, there is also a growing effort aimed at describing the mechanism by which the yeast mitochondria meet their metabolic requirement for flavin cofactors. Mitochondria that are isolated from *S*. *cerevisiae* are able to utilize exogenous riboflavin in the synthesis of FMN and FAD. This activity suggests these organelles contain both a riboflavin uptake system as well as riboflavin kinase and FAD synthetase enzymes [23]. Further analysis of this riboflavin-uptake-activity suggests that it is both concentration-dependent and biphasic, which is consistent with a model of at least two transport systems with differing affinities for their target [40].

Transport of FAD across the mitochondrial membrane in *S*. *cerevisiae* is believed to be facilitated by Flx1p [40,41], however, its exact role remains undefined. For example, work from Baffuno et al. indicates that Flx1p is an FAD exporter [40], however, this activity may be conditional as others have reported Flx1p mutants exhibit a decreased FAD content in mitochondria when *S*. *cerevisiae* are grown on the fermentable sugar galactose, consistent with the characteristics of a FAD importer [40,41]. Furthermore, yeast that contain Flx1p mutations show respiratory deficiency, which has been attributed in part to a decreased activity of succinate dehydrogenase and lipoamide dehydrogenase–both of which require FAD for activity [28,40,41]. The respiratory phenotype of the Δ*flx1* mutants can be rescued by overexpressing FAD1, although the specific mechanism for this effect has not been put forward [28]. Boone and colleagues (2010) determined that secondary deletion of the YMR178w ORF (whose function was unknown at the time) in *S*. *cerevisiae* may partially counteract a decrease in fitness that is observed by deletion of the *flx1* ORF [42]. The effect of the YMR178w downregulation on the Δ*flx1* mutant is similar to the effect of the FAD1 upregulation with respect to the FLX1 mutant, suggesting antagonistic functions of the two.

In this work, we report the cloning and characterization of the protein encoded by the YMR178w gene. The protein, hereby designated Fpy1p, was expressed in a recombinant system, purified, and characterized as a potassium-dependent pyrophosphatase with the capability of hydrolyzing FAD, NAD(H), and ADP-ribose. Fpy1p is unrelated to those of the NUDIX superfamily and represents a fully novel type of pyrophosphatase, with sequence homologs fused to FAD synthetases in animals and plants. Using this system, we additionally confirm the finding of Boone [42] that mutation of Fpy1p abolishes the decreased fitness that results from deletion of the FLX1 ORF. We also characterize a new negative genetic interaction between *fpy1* and the FMN biosynthetic gene *fmn1*, and present data suggesting a possible mechanism for this effect.

## Results

### Bioinformatic sequence analysis

Previous studies identified a positive interaction between the uncharacterized gene YMR178w (henceforth referred to as *fpy1*) and the gene encoding mitochondrial FAD transporter, *flx1* [42]. This activity led our research group to hypothesize that *fpy1* may play a role in the metabolism of flavin cofactors. Fpy1p has sequence homologs across multiple kingdoms of life. S1 Fig shows select sequence homologs of the *S*. *cerevisiae* protein in representative mammal, fish, invertebrate, algal, and dicot/monocot plant species.

Unlike the yeast Fpy1p, homologous proteins in higher eukaryotes are fused to an extra domain of approximately 250 residues. This extra domain is present on the C-terminus for animal homologs and on the N-terminus of plant proteins, which is consistent with the occurrence of two separate fusion events in evolutionary history. This extra domain is homologous to Fad1p, the *S*. *cerevisiae* protein previously demonstrated to be an FAD synthetase [28]. FAD synthetase activity has also been shown for the human homolog [43,44]. These results are consistent with convergent evolution toward fusion of an FAD synthetase with an Fpy1p-like protein, providing additional support for the idea that Fpy1p is, like Fad1p and Flx1p, involved in metabolism of flavin cofactors.

### Cloning and recombinant expression of FPY1p

The ORF encoding *S*. *cerevisiae* Fpy1p was amplified by PCR from the vector supplied by Open Biosystems. The resulting DNA fragment was subcloned into pDONR221, then Gateway-cloned into pYES-DEST52, followed by transformation and functional expression in *S*. *cerevisiae*.

### Purification, native molecular weight determination, and biochemical characterization of FPY1p

The recombinant FPY1p was designed to include a V5 epitope at the C-terminus as well as a polyhistidine (6xHis) tag for two-step purification by IMAC chromatography. Separation by SDS-PAGE demonstrated high purification of a protein of ~35 kDa (S1 Fig), which is consistent with the theoretical molecular weight for Fpy1p (35.4 kDa), as calculated from the amino acid sequence. Gel filtration chromatography was then used to estimate the molecular weight of native Fpy1p, which eluted in a well-defined peak with a molecular weight of 102–111 kDa. Because this value is three times the theoretical molecular weight of 35.4 kDa we, therefore, conclude that Fpy1p is active as a trimer.

**Fig 1:**
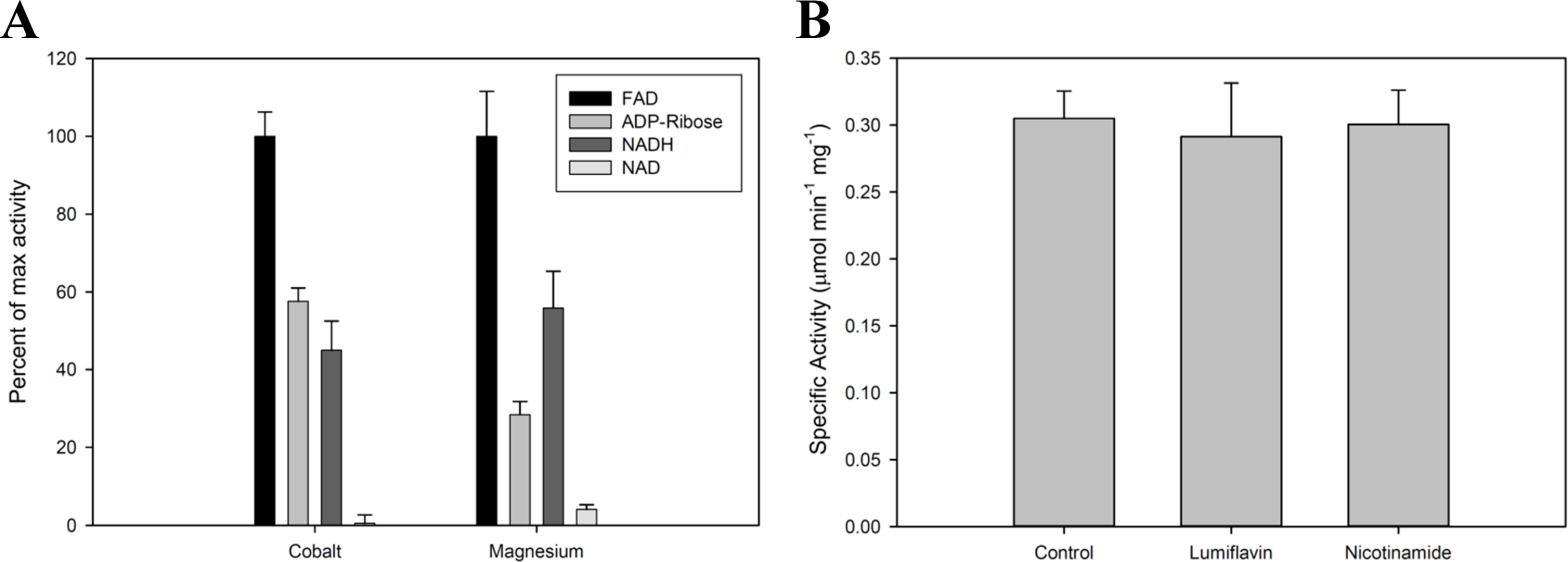
Analysis of substrate binding of Fpy1p. (A) Substrate specificity of Fpy1p. Data are expressed as a percent of activity relative to that observed when FAD is used as substrate with each metal ion, and is given as the average ± S.E. of three triplicate determinations in which AMP production was quantified. Assays were performed with 100 μM substrate along with either 4 mM CoCl_2_ (cobalt) or 10 mM MgCl_2_ (magnesium). (B) FAD pyrophosphatase activity of Fpy1p in the presence of potential inhibitors. Data are shown as the average ± S.E. of three triplicate determinations in which FMN production was quantified. Assays were performed using 4 mM CoCl_2_, 20 μM FAD, and 50 μM lumiflavin or nicotinamide when specified. Higher concentrations were not used due to limited solubility of lumiflavin.

Recombinant Fpy1p exhibits the ability to hydrolyze FAD, and other substrates with similar structures (Fig 1A) such as ADP-ribose and NADH; however these were hydrolyzed at about half the rate of FAD. Interestingly, while considerable hydrolysis is observed with NADH as the substrate, little or no activity was detected when using NAD^+^. The only substantial difference between the two molecules is the presence of a positive charge at the nicotinamide moiety, indicating electrostatic interactions may interfere with substrate binding. Additionally, when assays included free nicotinamide or lumiflavin as potential competitive inhibitors, the reaction was unaffected (Fig 1B), suggesting that these moieties are unable to bind the enzyme as lone entities. Therefore, it is likely that steric effects, rather than direct binding, determine the substrate specificity. Further characterization of the biochemical properties of Fpy1p was conducted using FAD because Fpy1p exhibited the highest activity with this substrate.

**Fig 2:**
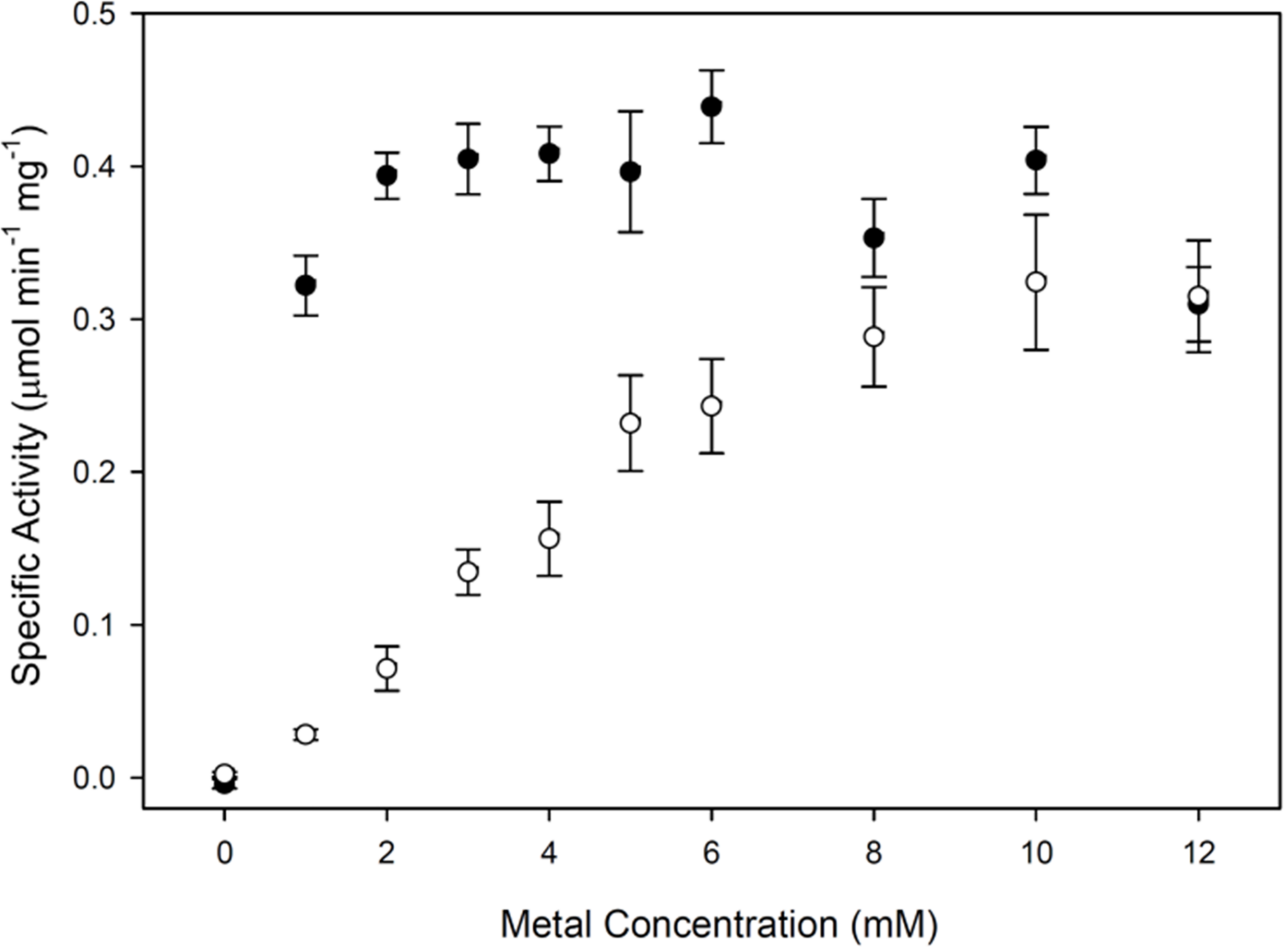
FAD pyrophosphatase activity of Fpy1p as a function of metal ion concentration. Data are shown as the average ± S.E. of three triplicate determinations. Assays were performed using either CoCl_2_ (solid circles) or MgCl_2_ (open circles) in the presence of 50 μM FAD.

Fpy1p was then assayed for changes in FAD pyrophosphatase activity when in the presence of a variety of metal activators. Significant enzymatic activity was observed with both Co^2+^ and Mg^2+^ (Fig 2), which occurred in a concentration-dependent manner: Co^2+^ yielded maximum activity at 2 to 6 mM whereas Mg^2+^ peaked at 8 to 12 mM. These maxima, though high relative to *in vivo* levels, are consistent with previous *in vitro* observations of enzymes with similar activities [39,45,46], and therefore 4 mM CoCl_2_ or 10 mM MgCl_2_ were used in our subsequent analyses. Both metals are accumulated by living organisms, and magnesium concentrations are generally higher than cobalt for yeast, though the specific concentrations vary among strains, the life cycle stages, and the environmental conditions [47]. Therefore, these results do not suggest whether one metal might be more relevant to the physiological function than the other.

**Fig 3:**
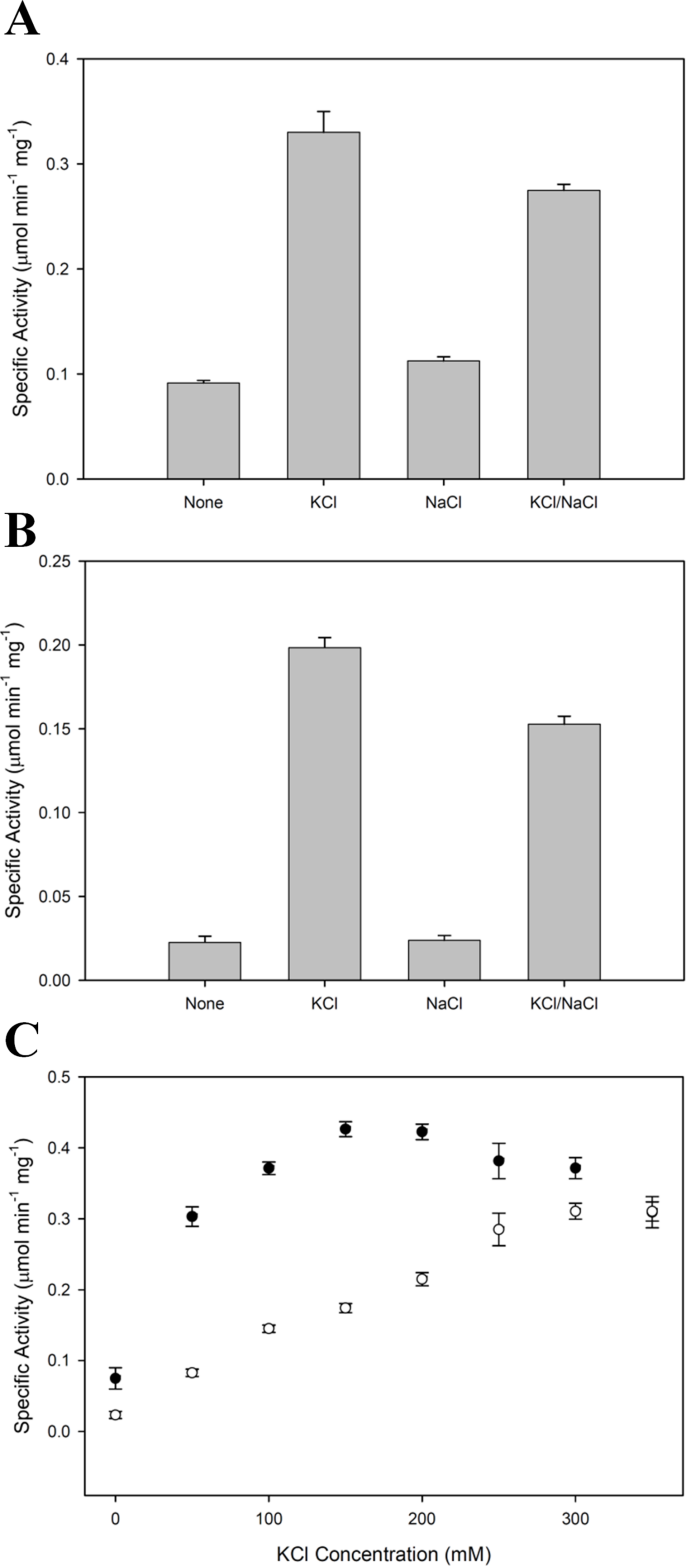
Potassium requirement for the FAD pyrophosphatase activity of Fpy1p. (A) Enzyme activity in the absence or presence of 200 mM KCl and/or 200 mM NaCl when assayed with 4 mM CoCl_2_. (B) Enzyme activity in the absence or presence of 200 mM KCl and/or 200 mM NaCl when assayed with 8 mM MgCl_2_. (C) Enzyme activity as a function of KCl concentration in the presence of 4 mM CoCl_2_ (solid circles) or 10 mM MgCl_2_ (open circles). Data are given as the average ± S.E. of three triplicate determinations.

The activation of Fpy1p by both Co^2+^ and Mg^2+^ was dependent on the co-presence of potassium ions, provided here as KCl (Fig 3). Sodium was unable to substitute for potassium, indicating the role of potassium is not generalizable to small monovalent cations, nor is it due to solely to an increase in ionic strength. Fpy1p required slightly higher concentrations of potassium for maximum activity when in the co-presence of magnesium as compared to cobalt. This difference may reflect a role of the potassium ions in facilitating the enzyme–metal–substrate interaction. For both cobalt and magnesium, maximum enzymatic activity was observed near the expected intracellular potassium concentration of 200 to 300 mM [48], thus suggesting that potassium dependence may not have a major effect on physiological function.

**Fig 4:**
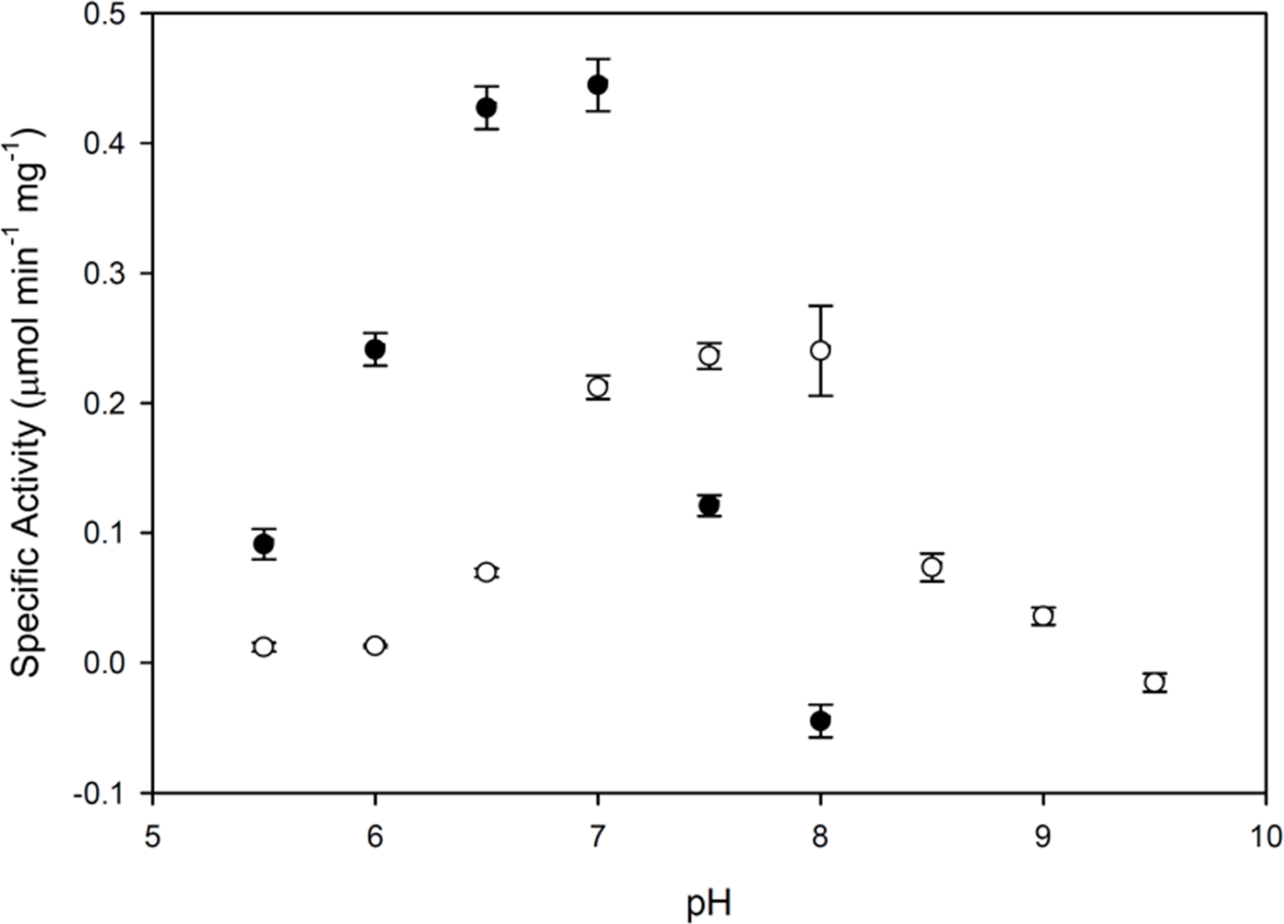
Effect of pH on FAD pyrophosphatase activity of Fpy1p. The enzymatic activity of Fpy1p was determined as described under *Experimental Procedures*, with the exception that MHC-KOH buffer (100 mM MES, 100 mM Hepes, 100 mM CHES) was used in place of HEPES-KOH. Assays included 50 μM FAD and either 4 mM CoCl_2_ (solid circles) or 10 mM MgCl_2_ (open circles). Data are given as the average ± S.E. of three triplicate determinations.

Cobalt and magnesium ions were also found to differ in their ability to activate Fpy1p as a function of pH (Fig 4). Whereas cobalt yielded maximal pyrophosphatase activity at pH 7.0, the presence of magnesium favored more alkaline conditions of pH 7.5–8.0. Some of this effect is likely due to the tendency of FAD to interact with some divalent metal cations and form precipitates at slightly alkaline pH [49]. Furthermore, the solubility of Co^2+^ is anticipated to decrease in aqueous solutions at alkaline pH due to the formation of cobalt(II) hydroxide [50], which would decrease the available Co^2+^ in solution. Therefore, the higher pH that was optimal with magnesium likely reflects the true pH optimum of the enzyme.

Fpy1p was found to follow standard Michaelis-Menten kinetics when specific activity was plotted as a function of FAD concentration (S2 Fig). The K_m_ was calculated as 16.1 ± 2.1 μM and 19.0 ± 2.7 μM in presence of Co^2+^ and Mg^2+^, respectively, while the V_max_ values were 0.641 ± 0.027 μMol min^-1^ mg^-1^ and 0.34 ± 0.027 μMol min^-1^ mg^-1^, respectively. Table 1 shows the kinetic properties of other enzymes that can hydrolyze FAD. While the K_m_ values vary considerably from enzyme-to-enzyme, ATNUDX23 (*A*. *thaliana*), the only pyrophosphatase verified to affect flavin homeostasis *in vivo*, has a K_m_ of 9.1 ± 0.9 μM and k_cat_ of 0.08 s^-1^ [31], which approximates what was observed for Fpy1p.

**Table 1:** Properties of FAD Purophosphatases as described in published reports.

Enzyme	Species	Metal tested	K_m_ (μM)	K_cat_ (s^-1^)	K_cat_/K_m_ (s^-1^ M^-1^)	Reference
Fpy1p	*S*. *cerevisiae*	4 mM Co^2+^	16.1 ± 2.1	0.38	2.3 x 10^4^	This work
Fpy1p	*S*. *cerevisiae*	8 mM Mg^2+^	19.0 ± 2.7	0.20	1.1 x 10^4^	This work
AtNUDX23	*A*. *thaliana*	5 mM Mg^2+^	9.1 ± 0.9	0.08	8.4 x 10^3^	[31]
YZGD	*P*. *thiaminolyticus*	10 mM Mn^2+^	3700 ± 600	22	6.0 x 10^3^	[36]
nudE.1	T4 bacteriophage	5 mM Mg^2+^	1050 ± 70	28	2.7 x 10^4^	[37]
Not cloned	*H*. *sapien*	25μM Co^2+^	12	1.4^a^	1.2 x 10^5^^a^	[34]
Not cloned	*P*. *radiates*	none^b^	16.5	nd^c^	nd^c^	[17]
Not cloned	*R*. *muridae*	none^b^	125	nd^c^	nd^c^	[35]

^a^Calculated based on reported V_max_ of 1.1 μmol min^-1^ mg^-1^ and enzyme molecular weight of 74,000 daltons.

^b^Authors did not report adding metal ions to assays for determination of K_m_ of these enzymes. However, it was found that the P. radiates enzyme was stimulated by inclusion of 10 mM Mg^2+^, whereas 1 mM Mg^2+^ had no effect, 1 mM Zn^2+^ stimulated, and 1mM EDTA inhibited the rat enzyme.

^c^Not determined for incompletely purified enzymes

### Analysis of mutant growth phenotypes

**Fig 5:**
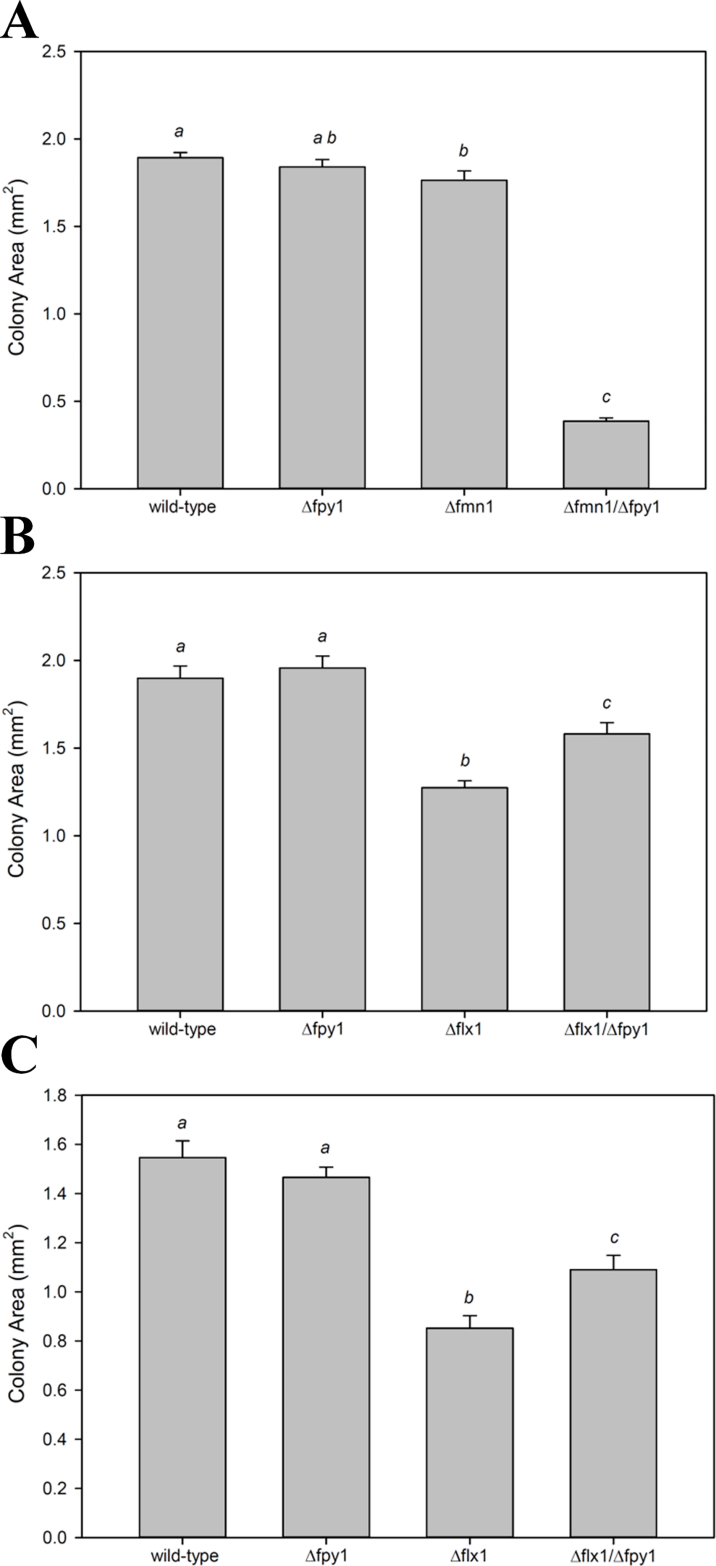
Growth analysis of wild type and mutant yeast under different conditions. (A) Colony size for wild type yeast and the Δ*fpy1*, Δ*fmn1*, and Δ*fmn1*/Δ*fpy1* mutants grown on YPD supplemented with 4 mM FMN for 48 hours. (B) Wild type yeast and the Δ*fpy1*, Δ*flx1*, and Δ*flx1*/Δ*fpy1* mutants grown on either SD/MSG medium for 72 hours or (C) YPD medium for 48 hours. Colony size was measured as described in *Experimental Procedures*. Data are given as the average ± S.E of 6–18 colonies per mutant line per condition tested. Different letters within each chart signify data which are statistically different (p < .05) based on one-way ANOVA with Holm-Šidák pairwise comparison.

To better understand the physiological role of Fpy1p, a yeast strain with a gene deletion in *fpy1* was acquired and the gene for *fmn1*, which encodes the dual-localized cytosolic/mitochondrial riboflavin kinase, was deleted in both Δ*fpy1* mutants and the wild-type parent yeast. In both backgrounds, the Δ*fmn1* mutation resulted in FMN auxotrophy, similar to what was previously described [27]. However, we additionally found a strong negative interaction between the two genes that has not been reported. Here the Δ*fmn1* single mutant displays only a 6–7% reduction in growth relative to wild-type when grown on media supplemented with 4 mM FMN (p<0.05), and the growth of Δ*fpy1* is statistically indistinguishable from that of wild-type, however, the Δ*fmn1*/Δ*fpy1* double mutant displays an 80% reduction in growth relative to wild-type under the same conditions (Fig 5A).

Additionally, a yeast strain with gene deletion of *flx1*, the mitochondrial FAD transporter, was acquired, then crossed with the Δ*fpy*1 strain to create a double mutant. Comparing the growth of yeast deletion mutants Δ*fpy1*, Δ*flx1*, and Δ*flx1*/Δ*fpy1* to wild-type for a variety of conditions demonstrated that colony size was greatly reduced for Δ*flx1* but not for *Δfpy1* when grown on agar plates (Fig 5). The Δ*flx1*/Δ*fpy1* mutant had increased colony size relative to the Δ*flx1* single mutant, demonstrating a positive genetic interaction between *flx1* and *fpy1*. This genetic interaction does not appear to be nutrient-dependent for colony formation and occurred similarly on both SD/MSG medium as well as rich YPD medium.

**Fig 6:**
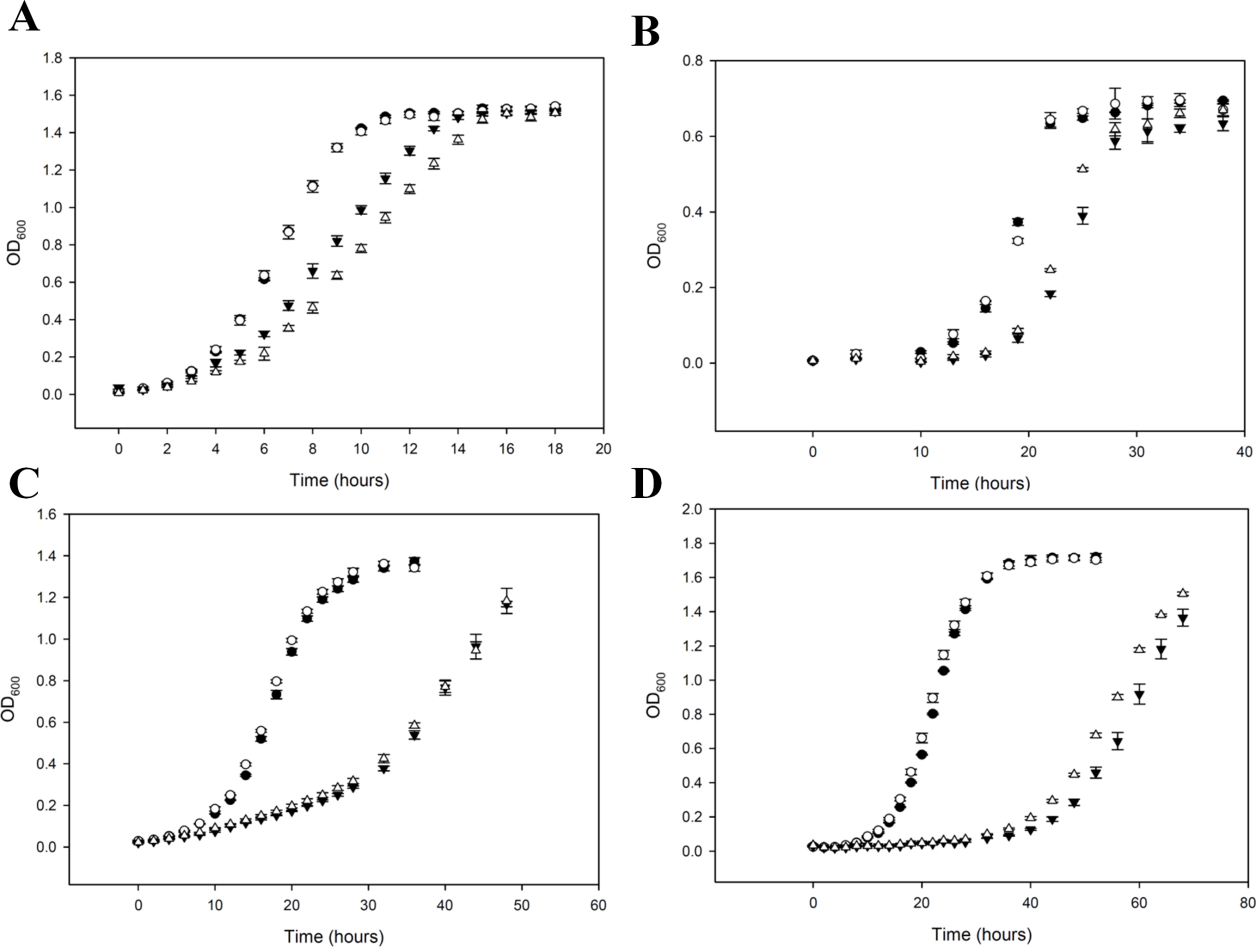
Growth of wild-type and mutant yeast under different conditions. The OD_600_ values were measured in a microplate (path length ~0.6 cm). Yeast were grown by flask culture in YP medium (1% yeast extract, 2% peptone) supplemented with 2% glucose (A, B), 3% glycerol (C); and 1% sodium acetate (D), under either normal (A, C, D) or hypoxic (B) conditions. Data are given as the average ± S.E. of three separate cultures. Symbols are: solid circles, wild-type; open circles, Δ*fpy1*; solid triangles, Δ*flx1*; open triangles, Δ*flx1*/Δ*fpy1*.

This positive interaction, however, is not evident when mutants are grown in liquid cultures under standard conditions and use glucose as the carbon source. Here, there is a slight decrease in fitness in the Δ*flx1*/Δ*fpy1* double mutant relative to the Δ*flx1* mutant alone (Fig 6A). However, when grown under hypoxic conditions with glucose as the carbon source, a weak positive interaction becomes evident (Fig 6B), similar to what was observed on colony plates. We, therefore, hypothesized that the positive synergy that was observed for the Δ*flx1*/Δ*fpy1* double mutant on colony growth plates is likely due to hypoxia, which can be induced by a dense colony environment.

The slow growth phenotype of the single Δ*flx1* mutant was more severe when grown on the non-fermentable carbon sources glycerol and acetate as compared to growth on glucose alone. This activity may be because production of NADH from those sources depends on mitochondria. Mitochondria need FAD for this requirement to be met, however, the Δ*flx1* mutant has less FAD in mitochondria than the wild type [28].

**Fig 7:**
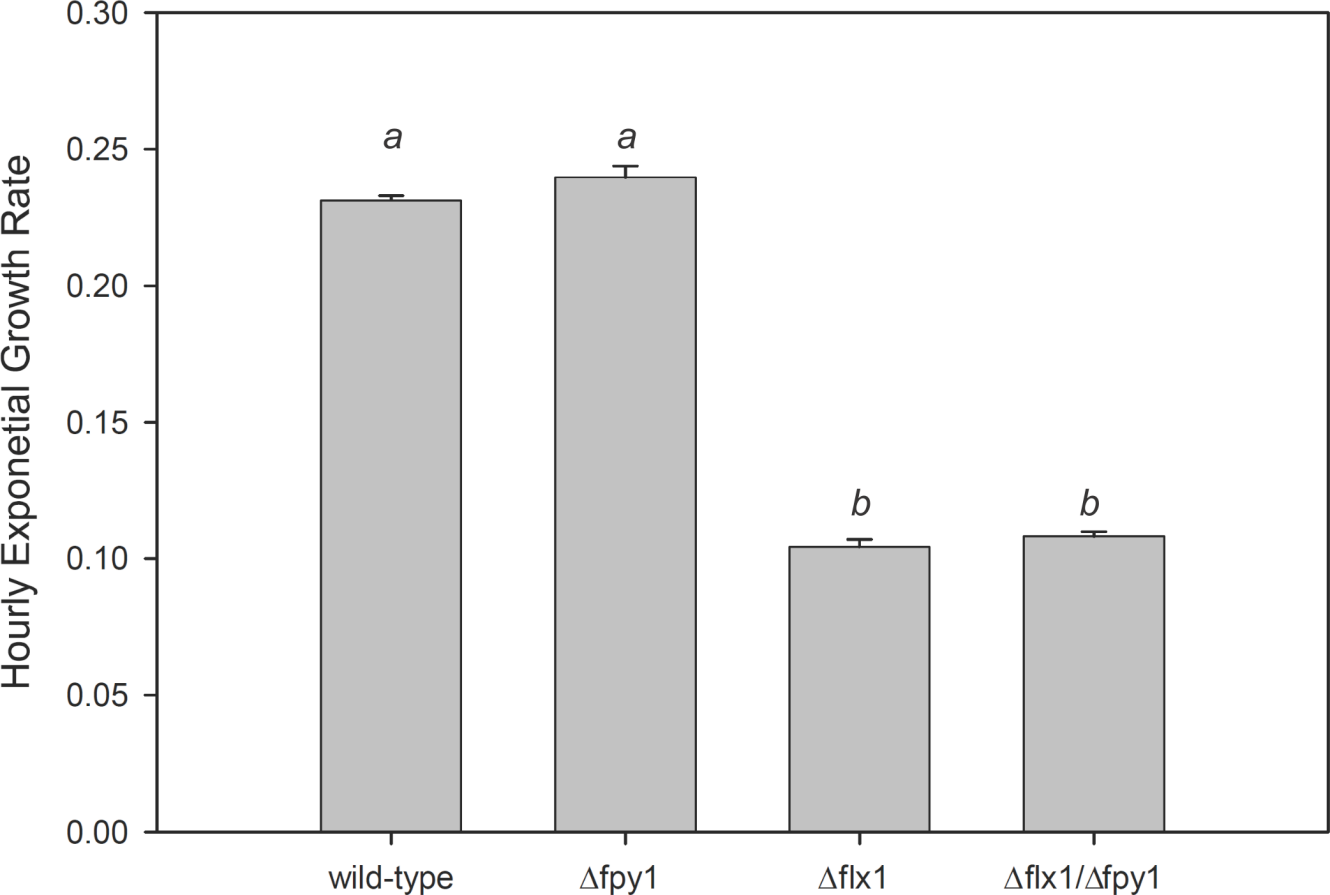
Comparison of exponential growth rates of yeast grown on YP + 1% sodium acetate. The portion of the growth curves which appeared linear on a semi-log plot (6–20 hours for wild type and Δ*fpy1*, 36–52 hours for Δ*flx1* and Δ*flx1*/Δ*fpy1*) were fit by least squares regression to the equation for exponential growth. Data for each condition are given as the average ± SE of three individual cultures. Different letters signify data which are statistically different (p < .05) based on one-way ANOVA with Holm-Šidák pairwise comparison.

A positive genetic interaction between *flx1* and *fpy1* is not evident when the yeast are grown on glycerol, which is non-fermentable and highly reduced (Fig 6C). However, when grown using the non-fermentable carbon source acetate, a weak positive genetic interaction between *flx1* and *fpy1* is again evident (Fig 6D). Analysis of the growth curves indicates that the improvement in growth of the Δ*flx1*/Δ*fpy1* double mutant over the Δ*flx1* single mutant occurs during either the lag phase or the lag/log transition, as the rate of logarithmic growth is statistically indistinguishable between the two strains (Fig 7).

### Activities of the mitochondrial FAD-dependent enzymes

Our results confirm previous observations [40,41] that Δ*flx1* mutants exhibit decreased activity of the mitochondrial FAD-dependent enzymes succinate dehydrogenase and lipoamide dehydrogenase regardless of which carbon source is present in the growth medium (Table 2). However, we found no significant effect on the activity of either enzyme in the Δ*fpy1* mutants, either alone or in combination with the Δ*flx1*/Δ*fpy1* background. Additionally, we noted an increase in the activity of mitochondrial alcohol dehydrogenase in the Δ*flx1* mutants (Table 2). This finding is consistent with the cells compensating for the loss of NADH-consuming enzymes within the mitochondria, as described in the *Discussion*.

**Table 2:** Results of mitchondrial enzyme assays. Mitochondria were isolated from yeast grown in YP medium supplemented with the listed carbon source. Specific activity is for total mitochondrial protein. Data is average ± SE of triplicate assays performed on single mitochondrial preparations. All assays performed as described in *Experimental Procedures*.

Carbon Source	Line	Specific Activity (μmol min^-1^ mg^-1^)		
		Succinate Dehydrogenase	Lipoamide Dehydrogenase	Alcohol Dehydrogenase
	WT	0.106 ± 0.007	0.433 ± 0.011	0.474 ± 0.022
Galactose	Δ*fpy1*	0.112 ± 0.003	0.436 ± 0.010	0.524 ± 0.009
	Δ*flx1*	0.013 ± 0.001	0.079 ± 0.002	0.701 ± 0.017
	Δ*flx1*/Δ*fpy1*	0.012 ± 0.001	0.110 ± 0.015	0.723 ± 0.038
	WT	0.246 ± 0.009	0.377 ± 0.008	1.070 ± 0.081
Glycerol	Δ*fpy1*	0.240 ± 0.010	0.444 ± 0.012	0.845 ± 0.028
	Δ*flx1*	0.035 ± 0.008	0.381 ± 0.008	1.802 ± 0.085
	Δ*flx1*/Δ*fpy1*	0.030 ± 0.001	0.322 ± 0.001	1.758 ± 0.018
	WT	0.247 ± 0.004	0.510 ± 0.011	0.660 ± 0.036
Acetate	Δ*fpy1*	0.245 ± 0.006	0.566 ± 0.020	0.757 ± 0.027
	Δ*flx1*	0.038 ± 0.006	0.347 ± 0.013	1.089 ± 0.005
	Δ*flx1*/Δ*fpy1*	0.032 ± 0.006	0.372 ± 0.012	0.914 ± 0.026

### Protein-protein interactions

As noted above, many eukaryotic species express a homolog of FPY1p as a fusion to a homolog of the yeast FAD synthetase, FAD1p. Because of this homology, we assayed whether the absence of this fusion is substituted in *S*. *cerevisiae* by a physical interaction, as well as whether the observed genetic interaction between *fpy1* and *flx1* might similarly be due to a physical interaction. To test this hypothesis, Fpy1p, Fad1p and Flx1p were expressed with fusions that, upon interaction of bait–prey proteins, an enhanced resistance to the cytotoxic agent methotrexate would be evident; however, no such resistance was observed, indicating no physical interaction between Fpy1p and either Fad1p or Flx1p proteins.

### Gene expression and FAD hydrolysis in the mutants

Despite the genetic interaction between *fpy1* and *flx1*, deletion of the *fpy1* gene did not result in a detectable change in expression of *flx1*, as determined by qRT-PCR (S3 Fig). Likewise, expression of *fmn1* and *fad1*, two other genes known to be involved in flavin cofactor interconversion, were not altered by deletion of *fpy1*. These results indicate a lack of *fpy1* expression is not subsequently compensated for by altering the expression pattern of other genes involved in flavin metabolism. Furthermore, mutating *fpy1* did not affect the total activity of yet-unidentified mitochondrial enzymes that hydrolyze FAD (S3 Fig).

### Flavin and NAD(H) contents in the yeast cells grown on acetate

To determine whether the observed growth defect of Δ*flx1* mutants and subsequent partial recovery in Δ*flx1*/Δ*fpy1* double mutants might be due to altered homeostasis of flavins or NAD(H), the abundance of these metabolites was determined in total cell extracts of stationary phase yeast grown on acetate (Table 3). The stationary cells were used because analysis of the growth curve showed that the effect on growth is a consequence of a delay during the transition of stationary cells into the log phase (Fig 7). We found that while the content of both flavins (FAD in particular) and nicotinamides are decreased in the Δ*flx1* mutant, the opposite effect is observed in the Δ*fpy1* mutant. This phenotype is consistent with previous reports that the Δ*flx1* mutant is susceptible to respiratory defects, and with FAD/nicotinimides serving as a substrate for FPY1 *in vivo*. Significantly, the net effect in the *Δflx1/Δfpy1* double mutant is an increase in flavins/nicotinamides over the levels observed in the Δ*flx1* mutant, suggesting that the reduction in hydrolysis of FAD and NADH in the double mutant is responsible for the compensation of the detrimental effects of Δ*flx1*, and the subsequent improvement in growth.

**Table 3:** Total cellular content of flavins and nicotinamides. Yeast were grown in liquid cultures with acetate as the carbon source. Metabolites were measured and described under “Experimental Procedures.” Data is the average ± SE of triplicate determinations.

	Nicotinamide content (nmol/g)			
Line	NAD^+^	NADH	NAD^+^:NADH	
WT	6.3 ± 0.2	2.6 ± 0.2	2.4 ± 0.2	
Δ*fpy1*	8.0 ± 0.2^a^	2.4 ± 0.4	3.7 ± 0.7	
Δ*flx1*	4.7 ± 0.4^a^	1.3 ± 0.1^b^	3.7 ± 0.3^a^	
Δ*flx1*/Δ*fpy1*	7.1 ± 0.6	1.9 ± 0.4	4.2 ± 0.9	
	Flavin content (nmol/g)			
Line	FAD	FMN	Riboflavin	FAD:FMN
WT	3.45 ± 0.04	1.65 ± 0.02	0.051 ± 0.017	2.10 ± 0.05
Δ*fpy1*	3.79 ± 0.13^a^	1.77 ± 0.04^a^	0.040 ± 0.004	2.14 ± 0.05
Δ*flx1*	2.84 ± 0.03^b^	1.21 ± 0.02^b^	0.054 ± 0.031	2.35 ± 0.04^a^
Δ*flx1*/Δ*fpy1*	3.26 ± 0.08^a^	1.58 ± 0.02^a^	0.028 ± 0.004	2.06 ± 0.02

^*a*^*p*<0.05

^*b*^*p*<0.005

## Discussion

A previous study identified YMR178w (now named Fpy1) as an enzyme of unknown function that may play a role in flavin nucleotide metabolism based on the genetic interaction with the mitochondrial FAD transporter Flx1p [42]. Analysis of the amino acid sequence for Fpy1p shows that this enzyme maintains sequence homologs across multiple kingdoms of life (S1 Fig). In plants and animals, Fpy1p homologs are fused to additional sequence homologs of yeast FAD synthetase Fad1p. The orientation of the two fused domains is reversed in plants versus animals (N- versus C- termini). Therefore, we conclude that the fusion in the two kingdoms arose from two different events in evolutionary history. This convergence suggests that an evolutionary advantage may be bestowed by linking the two proteins, and is consistent with our finding that Fpy1p acts as an FAD pyrophosphatase as fusion with an FAD synthetase would enable co-regulation of two opposing activities. Fusion of the opposing activities in flavin cofactor metabolism has been reported previously in plants in the bifunctional enzyme AtFMN/FHy from *A*. *thaliana* [14].

While several enzymes have been cloned that are capable of hydrolyzing FAD, to our knowledge all are members of the Nudix superfamily of hydrolases [31–33,36,37]. The members of this superfamily, of which there are six encoded by the *S*. *cerevisiae* genome, possess a conserved 23-amino acid consensus sequence referred to as the Nudix box [38]. Fpy1p, however, lacks the Nudix box and also any general sequence similarity to enzymes of the Nudix superfamily, making it a new type of FAD pyrophosphatase. Despite lacking the Nudix box, the activity of Fpy1p resembles that of members of the Nudix superfamily in that it is capable of hydrolyzing an array of nucleotide diphosphate substrates. Also similar to the activity of Nudix proteins, Fpy1p has a clear dependence on the presence of a metal cofactor. Our studies indicate that magnesium and cobalt both support the catalytic activity of Fpy1p, although higher concentrations of magnesium are required than of cobalt to achieve a similar level of activity. As summarized in Table 1, both metals also support the catalytic activity of FAD pyrophosphatases from diverse organisms. Magnesium and cobalt could be relevant to FAD metabolism, as the FAD synthetase activity of the human homolog of Fad1p was detected at high levels with both cobalt and magnesium [51]. Unlike NUDIX proteins, Fpy1p has a requirement for potassium, a requirement which cannot be fulfilled by other small cations such as sodium. The exact nature of this requirement is unknown, but similar reports have been given for a pyrophosphatase from prokaryotes as well [39].

A previous study that used GFP fusion concluded that Fpy1p localizes to both the cytosol and nucleus [29]. Since the authors report mislocalization of known cytosolic enzymes to the nucleus in their study, and Fpy1p lacks any known nuclear localization tag, it is likely that this protein is cytosolic. Additionally, since the nuclear pore complex is freely permeable to metabolites [52], any protein targeted to the nucleus would act on the same pool of FAD substrate.

Consistent with a role in FMN homeostasis, we found a strong negative genetic interaction between *fpy1* and the riboflavin kinase encoding gene *fmn1*. Whereas growth of the Δ*fmn1* deletion mutants can be restored to near wild type levels by supplementation with exogenous FMN [27] the deletion of *fpy1* in the Δ*fmn1* background reduces colony growth by 78% despite no discernible effect of the *fpy1* deletion in the wild type background (Fig 5). Such a negative interaction is strong evidence for complementary function [42]. Thus, these results demonstrate that, in addition to the two previously reported processes that generate intracellular FMN–phosphorylation of riboflavin via FMN1p and uptake from the environment–hydrolysis of FAD via FPY1p is also a physiologically relevant mechanism for maintaining FMN homeostasis. Furthermore, we confirmed the genetic interaction previously reported between FLX1 and FPY1 as evident by the improved colony growth of the double Δ*flx1*/Δ*fpy1* mutant as compared to the Δ*flx1* single mutant. This characteristic is evident on both defined MSG plates and YPD plates, confirming that the phenotype is not dependent on the media composition. This same effect is only seen in liquid culture when yeast are grown under hypoxic conditions, or if non-fermentable acetate is used as a carbon source. Analysis of the growth curves suggests that the effect is related to events occurring during the exit of the cells from the stationary phase. Measurements of flavins and NAD(H) reveal that both are increased in the double mutant as compared to the single mutant.

**Fig 8:**
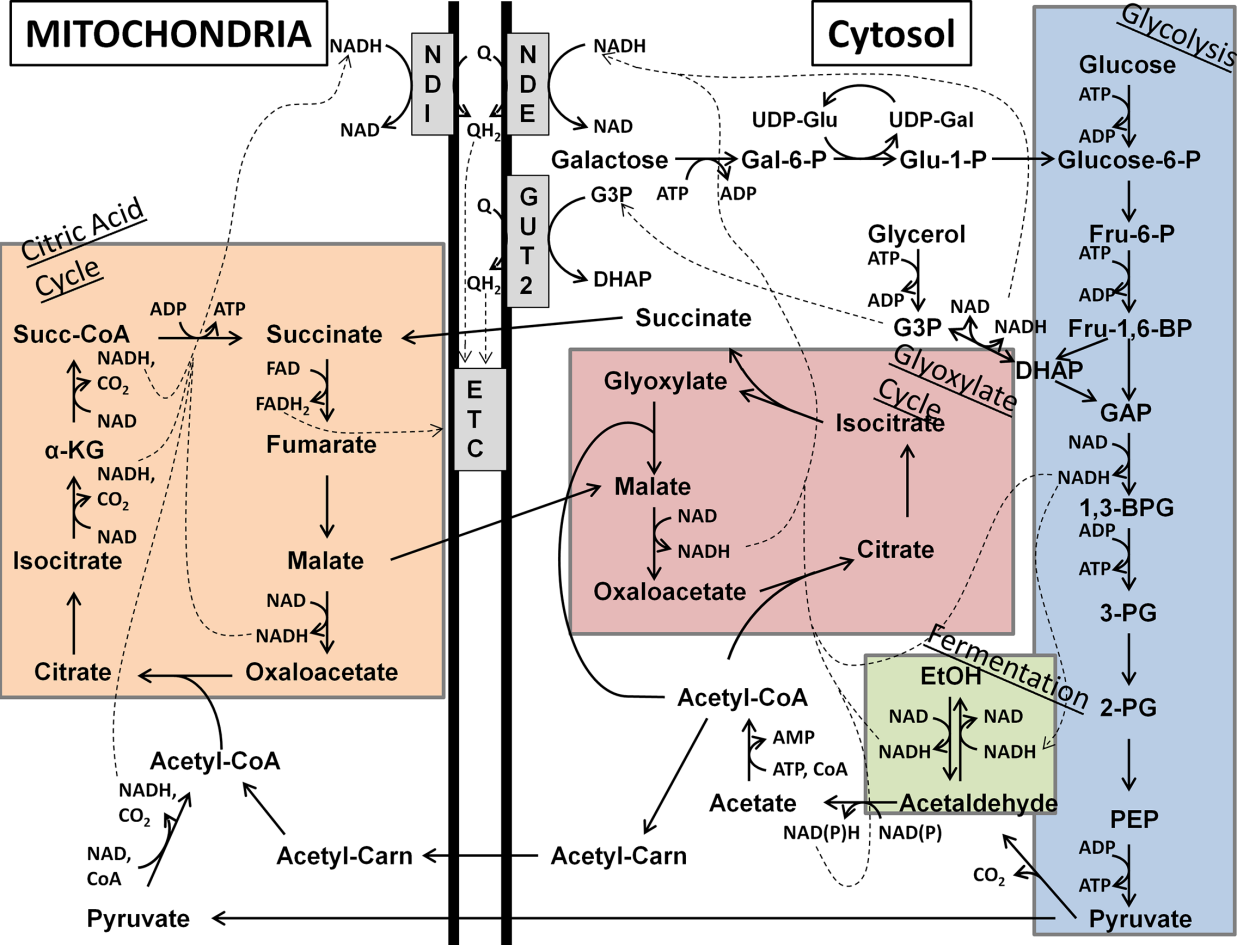
Known routes for production of NADH and subsequent contribution to mitochondrial electron transport chain from catabolism of various carbon sources. Included are glycolysis (blue), the glyoxylate cycle (rose), fermentation (green), and the citric acid cycle (orange), as well as the connecting steps between the pathways. Also shown are the routes for entry of galactose and glycerol catabolic products into the core glycolysis pathway. Abbreviations not described in the text are as follows: Fru-6-P, fructose-6-phosphate; Fru-1,6-BP, fructose-1,6-bisphophate; GAP, glyceraldehyde-3-phosphate; 1,3-BPG, 1,3-bisphosphoglycerate; 3-PG, 3-phosphoglycerate; 2-PG, 2-phosphoglycerate; PEP, phosphoenolpyruvate; Gal-6-P, galactose-6-phosphate; Glu-1-P, glucose-1-phosphate; UDP-Glu, UDP-glucose; UDP-Gal, UDP-galactose; Acetyl-Carn, acetylcarnitine; α-KG, alpha-ketoglutarate; Succ-CoA, succinyl-CoA; GUT2, mitochondrial G3P dehydrogrenase; ETC, electron transport chain.

The available evidence, in particular the decreased activity of mitochondrial enzyme activities as observed here and elsewhere [40,41], suggests that the Δ*flx1* mutants suffer from perturbation of the citric acid cycle and mitochondrial electron transport. The decrease in the mitochondrial FAD contents in the Δ*flx1* mutant perturbs the FAD-dependent mitochondrial metabolism, as shown by the reduced activities of the FAD-dependent enzymes lipoamide dehydrogenase and succinate dehydrogenase (citric acid cycle and the respiratory chain) [40,41], that were previously reported and confirmed here (Table 2). The reduced activity of these enzymes would cause perturbed energy production via the citric acid cycle. A reduced mitochondrial FAD concentration could also perturb function of the mitochondrial internal flavin-requiring NADH:ubiquinone oxidoreductase (NDI1), which is required for mitochondrial NADH to enter the electron transport chain. This disruption would further exacerbate the respiratory deficiency. Our new finding that the ADH activity is increased in these mutants suggests that the cells may be attempting to compensate for the defect in the citric acid cycle by increasing ethanol metabolism, which can be used by *S*. *cerevisiae* as a means of NADH mobilization [53]. In a previous study, the citric acid cycle was largely disabled by a mutation eliminating NDI1, the internal mitochondrial NAD reductase which is the entry point for electrons from citric acid cycle derived NADH into the mitochondrial electron transport chain [54]. As shown in Fig 8, this change required mitochondrial respiration to proceed entirely via the contribution of reduced NADH from the cytosolic face of the mitochondrial membrane via the external equivalents of NDI1, namely NDE1 and NDE2. Therefore, the mutated yeast could survive on carbon sources that were reduced enough to contribute sufficient cytosolic NADH for energy production, including the fermentable glucose, as well as non-fermentable glycerol. However, the mutant yeast were unable to grow on more oxidized carbon sources, such as acetate, presumably because these sources did not contribute sufficient cytosolic NADH to compensate for the citric acid cycle defect in the mitochondria, demonstrating the necessity of the citric acid cycle for energy production under these conditions. It has further been shown that a second FAD-dependent route for entry of cytosolic reducing equivalents into the electron transport chain, the glycerol-3-phosphate (G3P) shuttle, acts additively with the function of the NDEs, albeit with a lesser contribution as shown by growth effects in mutants [55].

Similar to the NDI1-deficient mutant, the flx1 mutant has a more severe phenotype when grown on acetate then when grown on glucose, likely because of the defective the citric acid cycle in mitochondria, as described above for the NDI1 mutant. Our data suggest that the fpy mutation helps alleviate this phenotype because reduced hydrolysis of FAD and NAD(H) in the mutants lacking Fpy1p in the cytosol helps in two ways: (1) by preserving the FAD needed by the cytosolic NADH-producing dehydrogenases and the G3P shuttle, and (2) by preserving the NADH produced in the cytosol. Thus, our data suggest that the role of Fpy1, a novel and non-Nudix pyrophosphatase, may be coordinating the regulation of FAD and NAD(H) contents in response to metabolic needs of the cell during the stationary phase.

## Materials and methods

### Materials

Except as otherwise noted, all chemicals and custom PCR primers were obtained from Sigma Aldrich. YeastBuster reagent was obtained from Novagen (Madison, WI). Yeast–Ura dropout supplement was obtained from Clontech (Mountain View, CA). Synthetic Drop-out Mix, minus Lysine, Methionine was obtained from US Biological (Swapscott, MA). FMN that was obtained from Sigma Aldrich was additionally purified as described previously [15].

### Bioinformatic analysis

Comparison of YMR178wp from *S*. *cerevisiae* with putative proteins from other species was conducted as follows: using the BLASTp suite available from NCBI (http://www.ncbi.nlm.nih.gov/), with YMR178wp as the query sequence, sequence homologs were identified in multiple species; for further analysis, we selected the homologs from a representative mammal (*Homo sapiens*, hFADS2), fish (*Danio rerio*, AAH80254), invertebrate animal (*Caenorhabditis elegans*, NP_001022287), green alga (*Chlamydomonas reinhardtii* XP_001693086), dicot plant (*Arabidopsis thaliana*, At5g03430), and monocot plant (*Oryza sativa*, OsI01253). Alignments were performed using ClustalW2 software (http://www.ebi.ac.uk/Tools/msa/clustalw2/) in conjunction with shading using BoxShade 3.21 (http://www.ch.embnet.org/software/BOX_form.html). Domain structure illustrations were created using DOG 2.0 [56].

### ORF cloning, vector construction, and expression in S. cerevisiae

The YMR178w open reading frame was acquired in vector BG1805 from Open Biosystems (Lafayette, CO). The open reading frame, excluding the stop codon, was amplified using *PfuTurbo* DNA polymerase (Stratagene, Santa Clara, CA) and the primer pair 5’-AAAAAAGCAGGCTTCATGGTGAAAGTAACTGCA-3’ (forward), and 5’-AGAAAGCTGGGTCGCTCTCCTGGTTCGAGAA-3’ (reverse). Note that the primers comprise both the Ymr178w-specific region (plain text), and the sequence that is needed for further downstream amplification by *attB* primers (underlined). The resulting PCR fragment was re-amplified using *attB* primers (Invitrogen), then separated by agarose gel electrophoresis, and purified using Wizard PCR columns (Promega, Madison, WI). The PCR product was sub-cloned by BP recombination into the pDONR221 vector (Invitrogen). The YMR178w open reading frame was transferred into the pYES-DEST52 destination vector from the pDONR221 construct via LR recombination. All cloning procedures were done in accordance with the manufacturer’s protocols. The constructs were verified by DNA sequencing.

The S.c. EasyComp Transformation Kit (Invitrogen) was used to generate chemically competent cells of the *S*. *cerevisiae* strain Y258 (Open Biosystems). The expression vector was transformed into these yeast following the manufacturer’s protocol. Yeast carrying the expression vector were cultured at 30°C in synthetic–Ura dropout medium (6.7 g/l yeast nitrogen base with ammonium sulfate, 0.77 g/l dropout supplement, 20 g/l raffinose). Once OD_600_ reached 1.0–1.2, 3x expression medium (30 g/l yeast extract, 60 g/l tryptone, 60 g/l galactose) was added at 50% of the culture volume, and incubation was continued for six hours before harvesting cells at 5000 × g and 4°C for 10 minutes.

### Recombinant protein isolation and native molecular weight determination

Extract of the *S*. *cerevisiae* cells expressing the recombinant protein was prepared by resuspending the cell pellet in 1x Yeastbuster with THP solution at a ratio of 5 ml of Yeastbuster per 1 g wet weight of cell pellet. After incubating at room temperature for 20 minutes with gentle shaking, the extracts were cleared by centrifugation at 16,000 × g, 4°C for 20 minutes. The recombinant proteins were purified from the cell lysate using an Äkta FPLC system equipped with 1-ml IMAC columns (GE Healthcare) charged according to the manufacturer’s protocol. All chromatography steps were performed at 4°C. A column charged with Cu^2+^ was equilibrated with Binding Buffer (50 mM potassium phosphate, pH 8.0, 500 mM KCl, 20 mM imidazole, 1 mM THP and 0.5% Tween-20) before loading the clarified cell lysates. Unbound proteins were removed by washing with 15 column volumes of Binding Buffer, followed by elution of bound proteins by a linear gradient of Binding Buffer to Elution Buffer (50 mM potassium phosphate, pH 8.0, 500 mM KCl, 500 mM imidazole, 1 mM THP, and 0.5% Tween-20) over 15 column volumes. Fpy1p eluted at ~125 mM imidazole. Fractions containing the desired protein were pooled and loaded directly onto a Ni^2+^-charged IMAC column equilibrated with Binding Buffer, which was then washed with 10 column volumes of Binding Buffer before elution with a linear gradient of Binding Buffer to Elution Buffer over 20 column volumes. The desired protein eluted at ~225 mM imidazole. Fractions containing Fpy1p were pooled and immediately desalted into Protein Storage Buffer (50 mM Hepes KOH, pH 7.0, 500 mM L-arginine HCl, 1 mM THP, 0.5% Tween-20) using Zeba™ Desalt Spin Columns (Thermo Scientific, Rockford, IL). Desalted samples were aliquoted and stored at -80°C until use.

Molecular weight of the native protein was estimated by gel filtration chromatography on a Superdex 200 10/300 column (GE Healthcare) equilibrated with gel filtration buffer (50 mM potassium phosphate, pH 7.5, 150 mM KCl, 100 mM L-arginine HCl, 0.1% Tween-20, and 1 mM THP). A calibration curve was generated using the following gel filtration standards from Sigma Aldrich: Cytochrome c (12.4 kDa), Carbonic Anhydrase (29 kDa), Bovine Serum Albumin (66 kDa), Alcohol Dehydrogenase (150 kDa), and β-Amylase (200 kDa). Standards were detected by measuring absorbance at 280 nm in 100-μl fractions collected after the chromatographic separation, while the Fpy1p activity was detected by enzymatic assay.

### Enzyme assays

Unless otherwise indicated, the procedures described below were used. Initial reaction rates at steady state were measured. Product formation was proportional to enzyme concentration and time. Less than 10% of the substrates were consumed in reactions. Assay buffer was 100 mM Hepes-KOH, pH7.0, 200 mM KCl, and 1 mM THP. Metal ions were added as described under the individual results. Assay volume was 25 μl.

Reactions were incubated at 30°C for 20 minutes, then quenched by addition of saturated formic acid to 5% of the assay volume. Precipitated protein was removed by centrifugation at 1500 x g for 15 minutes at 4°C. Reaction products and substrates were separated by reverse-phase chromatography using a Waters Alliance 2695 HPLC system with a Waters SunFire C_18_ column (4.6 × 150 mm, 3.5 μm) linked to a 2475 fluorescence detector, and were measured by fluorescence detection using an excitation wavelength of 470 nm and an emission wavelength of 530 nm. The mobile phase contained 100 mM ammonium formate, 100 mM formic acid, and 20% methanol. Product formation was determined from fluorescence by comparison to standards. FMN and FAD concentrations in standard solutions were determined spectrophotometrically [57]. Alternatively, the reaction product AMP was detected after derivatization at 80° C for 10 minutes in a final volume of 175 μl that contained the entire quenched reaction mixture, 3.6% chloroacetaldehyde, and citrate/phosphate (0.48 M and 0.59 M, respectively) buffer, pH 4.0 [58]. Derivatized samples were immediately cooled on ice, then centrifuged at 1500 x g, 4° C for 1 hour. Samples were separated by HPLC using a Waters XTerra MS C_18_ column (4.6 × 100 mm, 5 μm). Separation was performed using the gradient in S1 Table. Derivatized AMP was measured by fluorescence detection using an excitation wavelength of 280 nm and an emission wavelength of 410 nm. Enzymatic product formation was determined by subtracting a blank in which enzyme was added after incubation.

The K_m_ and k_cat_ values were determined by varying the substrate concentration. The results were fit to the Michaelis-Menten equation using non-linear regression analysis software of the Enzyme Kinetics Module 1.2 in SigmaPlot 9.0.

### Mutant strain development and growth

Haploid deletion mutant strains for fpy1 in a BY4742 background (MATα his3Δ1 leu2Δ0 lys2Δ0 ura3Δ0) and flx1 in a BY4741 background (MATa his3Δ1 leu2Δ0 met15Δ0 ura3Δ0) were acquired from Open Biosystems. Single colonies of each strain were picked from fresh YPD streak plates, patched together onto YPD agar, and incubated for 30° C to permit mating. After four hours, mated yeast were streaked onto selective SD/His/Ura/Leu plates (6.7 g/l yeast nitrogen base with ammonium sulfate, 20 g/l dextrose, 20 μg/ml histidine, 20 μg/ml uracil, 30 μg/ml leucine), and the resultant heterozygous double mutant were grown at 30° C. Sporulation and random spore analysis were performed as described previously [59], with the exception that a glucose-free sporulation medium was used (10 g/l potassium acetate, 20 μg/ml histidine, 20 μg/ml uracil, 30 μg/ml leucine). DNA was isolated from resultant colonies by the Bust n’Grab method [60], and used in PCR to identify the mutant genotype. Primer sequences were selected based on the recommendation of Open Biosystems (S2 Table). All identified strains were verified as haploid by their inability to sporulate using the method described above, and their mating type determined by observation under light microscopy for development of shmoos in the presence of either a known MATa or MATα strain. MATα strains for wild-type, Δ*fpy1*, Δ*flx1*, and Δ*flx1*/Δ*fpy1* were isolated. To generate Δ*fmn1* deletions, one-step gene replacement was employed in the wild-type and Δ*fpy1* MATα backgrounds. The nourseothricin-resistance gene (NourS) was amplified from plasmid pAG25 [61] using the primers 5’-CGTGAAGCGGTCACAGACACGTGTTGTTGAAGTGTTGATG CAGCTGAAGCTTCGTACGC-3’ and 5’-TAAGAAAAACTACTAGCCTCATCACTCCCGCAGATCTCTA GCATAGGCCACTAGTGGATCTG-3’, which include extensions (underlined) corresponding to the flanking sequences of *fmn1*. One step integration was performed to incorporate PCR products into the yeast genome to replace the *fmn1* gene with the nourseothricin-resistance gene following the previously published protocol [62]. The transformants were subsequently plated on YPD medium supplemented with 4 mM riboflavin 5’-monophosphate sodium salt hydrate (FMN-Na) (Sigma-Aldrich) and 200 mg/L nourseothricin to select for incorporation of the resistance gene. To verify the gene replacement, colonies surviving selection were subject to DNA extraction using the ZymoprepTM Yeast Plasmid Miniprep I kit (Zymo Research), and PCR verified by presence of an amplicon using NourS primers (5’-CAGGGGCATGATGTGACTGT-3’ and 5’- GTACTGATTAGGGGCAGGGC -3’) and absence of an amplicon with *fmn1*-specific primers (5’- AATACCTGCGCAACCAGGTC and 5’-CTTGACCCTGGCCCCATAAA-3’).

To determine the relative fitness of the mutants, single colonies of each strain were grown overnight in liquid YPD (with FMN supplementation where applicable) at 30° C with shaking. Overnight culture was diluted and spread on SD-MSG/His/Ura/Leu plates (0.71 g/l YNB-Potassium Phosphate [Sunrise Science, San Diego, CA] 1 g/l KH_2_PO_4_, 1 g/l L-glutamic acid, 20 μg/ml histidine, 20 μg/ml uracil, 30 μg/ml leucine, 15 g/l bacto agar) or YPD plates (with FMN supplementation where applicable). After 48 and 72 hours for the YPD and SD-MSG plates, respectively, digital photographs were taken, including grid paper for size reference. Using ImageJ software [63], the diameter of colonies was measured. Additionally, overnight cultures pregrown in YPD were used to inoculate liquid flask cultures of YP media with the appropriate carbon source (2% dextrose, 3% glycerol, or 1% sodium acetate) to OD_600_ of 0.05, 1 cm path length. Cultures were grown at 30° C with shaking, and OD_600_ measurements were taken at regular intervals. For growth under hypoxic conditions, YPD medium was saturated with N_2_ gas and flasks were sealed with robber stopper. Yeast cells were incubated at 30°C without agitation, and OD_600_ measured as above.

### Isolation of mitochondria

Mitochondria were isolated from liquid cultures of all strains at late log phase following a previously published method, except culture medium was altered as applicable [64].

### Mitochondrial enzyme assays

Mitchondria were disrupted by dilution of 50-μl mitochondrial suspension with 200 μl of 50 mM potassium phosphate, pH 7.8 containing 0.5% Tween-20, followed by three cycles of freezing and thawing. This suspension was assayed directly for succinate dehydrogenase activity using a previously published method [65] at 1.8 mM phenazine methosulfate, except 1 mM 8-hydroyquinoline was used in place of potassium cyanide, as it has previously shown to be suitable [66]. Disrupted mitochondria were centrifuged at 20,800 × g, 4° C, for 15 minutes and the supernatant desalted into 50 mM potassium phosphate, pH 7.8, using Zeba™ Desalt Spin Columns. The resultant protein suspension was assayed for lipoamide dehydrogenase according to published procedure [67], except lipoamide stock solutions were prepared in dimethylformamide to eliminate the substantial effect of alcohol dehydrogenase on NADH concentrations in the assay, encountered when stock solutions were prepared in ethanol. The same suspensions were assayed for alcohol dehydrogenase activity according to a published method [68]. Identically prepared mitochondrial protein suspensions were used in FAD pyrophosphatase assays as described above, except KCl was decreased to 150 mM, and HEPES-KOH pH 7.0 was replaced with potassium phosphate pH 7.8 to better reflect the pH of the mitochondrial matrix and the previously described pH optimum for mitochondrial FAD pyrophosphatase activity [26].

### Analysis of metabolite content

Flavin contents were measured using a procedure adapted from [69]. Yeast cultures were pelleted by centrifugation at 5000 × g, 4°C for 10 minutes. Pellets were resuspended in pure water and centrifuged again. Yeast pellets were then resuspended in flavin extraction solution (9:10 methanol:methylene chloride) at a ratio of 20 μl per milligram of cell paste. Nine parts 0.1 M ammonium acetate, pH 6.0 was added per 19 parts of flavin extraction solution and vortexed to mix. Samples were then centrifuged at 5000 × g at room temperature. Flavins in the upper, aqueous phase were passed through a 0.45 μm filter before being separated and quantified as described above for enzyme assays.

NAD and NADH contents were determined using the method adapted from a previously published protocol [70]. Briefly, 600 μl ammonium acetate (50 mM, saturated with N_2_ gas) was added to 200 μl mitochondrial suspension and vortexed to mix. Mitochondia were disrupted by five cycles of freezing and thawing, then centrifuged at 10,000 × g for 1 min at 4°C. The supernatant was saved, and the pellet was extracted in 600 μl of a N_2_-saturated acetonitrile and 50 mM ammonium acetate (3:1 v/v) before centrifugation at 10,000 × g for 5 min at 4°C. The resulting supernatant was pooled with the prior supernatant. Lipids were extracted with chloroform before the resulting aqueous phase from three preparations were pooled, snap frozen, and lyophilized for 15 h. Lyophilized samples were resuspended in 100 μl ice-cold 50 mM ammonium acetate, passed through a 0.45 μm filter, and analyzed by HPLC.

NAD and NADH were isolated from cytosolic supernatant as above with the following changes: 1.5 ml ammonium acetate (50 mM, saturated with N_2_ gas) was added to 200 μl cytosolic supernatant, vortexed to mix, and centrifuged at 10,000 × g for 1 min at 4°C. The remaining procedures were kept the same as above.

### Investigation of yeast protein-protein interaction

Yeast bait and prey strains were acquired from the Protein Interactome collection at Open Biosystems (Lafayette, CO). Individual interactions were tested using a method adapted from Tarassov et al [71]. Single colonies of a single bait strain and a single prey strain were picked from YPD plates, patched together on YPD agar, and incubated at 30° C for 4 hours to permit mating. To select for diploids, mated yeast were streaked onto selective SD–Lys–Met plates (6.7 g/l yeast nitrogen base with ammonium sulfate, 20 g/l dextrose, 2 g/l Synthetic Drop-out mix minus lysine, methionine) supplemented with 250 μg/ml hygromycin B and 100 μg/ml nourseothricin. To test for a physical interaction, the diploid strains were spotted onto SD–Lys–Met plates supplemented with 200 μg/ml methotrexate [72].

### RNA extraction and gene expression analysis

Wild-type and Δfpy1 yeast were grownhto log phase in YPD. Total RNA was extracted using the PureLink RNA Mini Kit (Ambion, Carlsbad, CA), following the manufacturer’s instructions for extraction by enzymatic disruption, and including the optional on-column Purelink DNase treatment. Reverse transcription was performed on 2 μg of RNA using Omniscript Reverse Transcription Kit (Qiagen, Hilden, Germany). The resulting cDNAs were diluted 1:10 in water and used in quantitative RT-PCR for determination of relative expression of *FAD1*, *FMN1*, and *FLX1*. Reference genes were *TFC1*, *ALG9*, and *UBC1*, based on previous recommendations [73]. Primers were designed using the Primer-BLAST tool from NCBI (S3 Table). Reactions consisted of 200 μM dNTP mix (Fermentas), 1x Platinum Taq Buffer, 2.5 mM MgCl_2_, 0.25x SYBR Green, 50 nM ROX, 0.5 units Platinum Taq DNA polymerase (all Invitrogen), and 0.25 μM custom primers from Sigma Aldrich. Real-time PCR was performed using an Applied Biosystems 7500 Real Time PCR System. Amplification was performed using a 7-min initial denaturation step at 95° C, followed by 40 cycles of a 15-s denaturation step at 95° C and a 1-min annealing/extension step at 60° C. All reactions were carried out in triplicate, and amplicon quality was analyzed using the melting curve function carried out by the Applied Biosystems 7500 PCR System. Relative expression was determined from the C_T_ values using the 2^-ΔΔCT^ method [74].

## Supporting information

S1 Fig (A) Multiple sequence alignment of Fpy1p from *S*. *cerevisiae* with select proteins from other organisms. The species’ abbreviations are for Sc, *Saccharomyces cerevisiae*; Hs, *Homo sapiens*; Dr, *Danio rerio*; Ce, *Caenorhabditis elegans*; Cr, *Chlamydomonas reinhardtii*; At, *Arabidopsis thaliana*; and Os, *Oryza sativa*. (B) Multiple sequence alignment of Fad1p from *S*. *cerevisiae* with same proteins as in (A). (C) Illustrated domain structures of the proteins in A and B. Blue denotes domains with sequence homology to Fad1p, while Red denotes domains with sequence homology to Fpy1p.
(TIF)

S2 Fig FAD pyrophosphatase activity of Fpy1p versus FAD concentration. Measurements were made in the presence of either (A) 4 mM CoCl_2_ or (B) 10 mM MgCl_2._ Data is the average ± S.E. of three triplicate determinations. Curve is nonlinear fit to the Michaelis-Menten model using SigmaPlot 9.0.
(TIF)

S3 Fig Relative expression of proteins involved in flavin metabolism in Δ*fpy1* deletion mutant. For both panels, solid black bar is wild-type control, solid grey bar is Δfpy1 deletion mutant. (A) Real-time PCR analysis of genes involved in flavin metabolism in yeast grown in YPD. Shown is the fold change relative to the housekeeping gene TFC1, calculated as described under “Experimental Procedures.” Data is the average ± S.E. of three technical replicates. (B) Mitochondrial FAD pyrophosphatase activity of yeast grown in YP supplemented with 1% sodium acetate or 3% glycerol. Assays were carried out as described under “Experimental Procedures” with 50 mM FAD and 10 mM MgCl_2_. Data is the average ± S.E. of three triplicate determinations.
(TIF)

S1 Table Gradient for separation of Derivatized AMP by HPLC. (DOCX)

S2 Table Primers for PCR verification of gene deletion. (DOCX)

S3 Table Primers for Real-time PCR. (DOCX)

## References

[1] Massey V (1995) Flavoprotein Structure and Mechanism Bethesda, MD, ETATS-UNIS: Federation of American Societies for Experimental Biology. 3 p. 10.1096/fasebj.9.7.7737454 7737454

[2] Fraaije MW, Mattevi A (2000) Flavoenzymes: diverse catalysts with recurrent features. Trends in Biochemical Sciences 25: 126–132. 10694883 10.1016/s0968-0004(99)01533-9

[3] De Colibus L, Mattevi A (2006) New frontiers in structural flavoenzymology. Curr Opin Struct Biol 16: 722–728. doi: 10.1016/j.sbi.2006.10.003 17070680 10.1016/j.sbi.2006.10.003

[4] Roje S (2007) Vitamin B biosynthesis in plants. Phytochemistry 68: 1904–1921. doi: 10.1016/j.phytochem.2007.03.038 17512961 10.1016/j.phytochem.2007.03.038

[5] Briggs WR, Olney MA (2001) Photoreceptors in plant photomorphogenesis to date. Five phytochromes, two cryptochromes, one phototropin, and one superchrome. Plant Physiol 125: 85–88. 11154303 10.1104/pp.125.1.85 PMC1539332

[6] Lin C, Todo T (2005) The cryptochromes. Genome Biology 6: 1–9. 10.1186/gb-2005-6-5-220 PMC1175950 15892880

[7] Bouly JP, Schleicher E, Dionisio-Sese M, Vandenbussche F, Van Der Straeten D, et al (2007) Cryptochrome blue light photoreceptors are activated through interconversion of flavin redox states. J Biol Chem 282: 9383–9391. doi: 10.1074/jbc.M609842200 17237227 10.1074/jbc.M609842200

[8] Bacher A, Eberhardt S, Fischer M, Kis K, Richter G (2000) Biosynthesis of vitamin b2 (riboflavin). Annu Rev Nutr 20: 153–167. doi: 10.1146/annurev.nutr.20.1.153 10940330 10.1146/annurev.nutr.20.1.153

[9] Bacher A, Eberhardt S, Eisenreich W, Fischer M, Herz S, et al (2001) Biosynthesis of riboflavin. Vitam Horm 61: 1–49. 11153262 10.1016/s0083-6729(01)61001-x

[10] Fischer M, Bacher A (2006) Biosynthesis of vitamin B2 in plants. Physiologia Plantarum 126: 304–318.

[11] Abbas CA, Sibirny AA (2011) Genetic control of biosynthesis and transport of riboflavin and flavin nucleotides and construction of robust biotechnological producers. Microbiol Mol Biol Rev 75: 321–360. doi: 10.1128/MMBR.00030-10 21646432 10.1128/MMBR.00030-10 PMC3122625

[12] Fischer M, Bacher A (2005) Biosynthesis of flavocoenzymes. Natural Product Reports 22: 324–350. doi: 10.1039/b210142b 16010344 10.1039/b210142b

[13] McCormick DB (1962) The Intracellular Localization, Partial Purification, and Properties of Flavokinase from Rat Liver. Journal of Biological Chemistry 237: 959–962.

[14] Sandoval FJ, Roje S (2005) An FMN Hydrolase Is Fused to a Riboflavin Kinase Homolog in Plants. Journal of Biological Chemistry 280: 38337–38345. doi: 10.1074/jbc.M500350200 16183635 10.1074/jbc.M500350200

[15] Sandoval FJ, Zhang Y, Roje S (2008) Flavin Nucleotide Metabolism in Plants. Journal of Biological Chemistry 283: 30890–30900. doi: 10.1074/jbc.M803416200 18713732 10.1074/jbc.M803416200 PMC2662166

[16] Yatsyshyn V, Fedorovych D, Sibirny A (2009) The microbial synthesis of flavin nucleotides: A review. Applied Biochemistry and Microbiology 45: 115–124. 19382698

[17] Kumar SA, Rao NA, Vaidyanathan CS (1965) Nucleotidases in plants: I. Partial purification and properties of the enzyme hydrolyzing flavine adenine dinucleotide from mung bean seedlings (Phaseolus radiatus). Archives of Biochemistry and Biophysics 111: 646–652. 5862212 10.1016/0003-9861(65)90246-8

[18] Byrd JC, Fearney FJ, Kim YS (1985) Rat intestinal nucleotide-sugar pyrophosphatase. Localization, partial purification, and substrate specificity. J Biol Chem 260: 7474–7480. 2987256

[19] Barile M, Brizio C, De Virgilio C, Delfine S, Quagliariello E, et al (1997) Flavin adenine dinucleotide and flavin mononucleotide metabolism in rat liver—the occurrence of FAD pyrophosphatase and FMN phosphohydrolase in isolated mitochondria. Eur J Biochem 249: 777–785. 9395326 10.1111/j.1432-1033.1997.00777.x

[20] Granjeiro JM, Ferreira CV, Jucá MB, Taga EM, Aoyama H (1997) Bovine kidney low molecular weight acid phosphatase: FMN-dependent kinetics. IUBMB Life 41: 1201–1208. 10.1080/15216549700202291 9161715

[21] Rawat R, Sandoval FJ, Wei Z, Winkler R, Roje S (2011) An FMN Hydrolase of the Haloacid Dehalogenase Superfamily Is Active in Plant Chloroplasts. Journal of Biological Chemistry 286: 42091–42098. doi: 10.1074/jbc.M111.260885 22002057 10.1074/jbc.M111.260885 PMC3234908

[22] Maruta T, Yoshimoto T, Ito D, Ogawa T, Tamoi M, et al (2012) An Arabidopsis FAD Pyrophosphohydrolase, AtNUDX23, is Involved in Flavin Homeostasis. Plant and Cell Physiology 53: 1106–1116. doi: 10.1093/pcp/pcs054 22505691 10.1093/pcp/pcs054

[23] Pallotta ML, Brizio C, Fratianni A, De Virgilio C, Barile M, et al (1998) Saccharomyces cerevisiae mitochondria can synthesise FMN and FAD from externally added riboflavin and export them to the extramitochondrial phase. FEBS Letters 428: 245–249. 9654142 10.1016/s0014-5793(98)00544-4

[24] Barile M, Brizio C, Valenti D, De Virgilio C, Passarella S (2000) The riboflavin/FAD cycle in rat liver mitochondria. Eur J Biochem 267: 4888–4900. 10903524 10.1046/j.1432-1327.2000.01552.x

[25] Giancaspero TA, Locato V, de Pinto MC, De Gara L, Barile M (2009) The occurrence of riboflavin kinase and FAD synthetase ensures FAD synthesis in tobacco mitochondria and maintenance of cellular redox status. FEBS Journal 276: 219–231. doi: 10.1111/j.1742-4658.2008.06775.x 19049514 10.1111/j.1742-4658.2008.06775.x

[26] Pallotta ML (2011) Evidence for the presence of a FAD pyrophosphatase and a FMN phosphohydrolase in yeast mitochondria: a possible role in flavin homeostasis. Yeast 28: 693–705. doi: 10.1002/yea.1897 21915900 10.1002/yea.1897

[27] Santos MaA, Jiménez A, Revuelta J (2000) Molecular Characterization of FMN1, the Structural Gene for the Monofunctional Flavokinase of Saccharomyces cerevisiae. Journal of Biological Chemistry 275: 28618–28624. doi: 10.1074/jbc.M004621200 10887197 10.1074/jbc.M004621200

[28] Wu M, Repetto B, Glerum DM, Tzagoloff A (1995) Cloning and characterization of FAD1, the structural gene for flavin adenine dinucleotide synthetase of Saccharomyces cerevisiae. Molecular and Cellular Biology 15: 264–271. 7799934 10.1128/mcb.15.1.264 PMC231949

[29] Huh W-K, Falvo JV, Gerke LC, Carroll AS, Howson RW, et al (2003) Global analysis of protein localization in budding yeast. Nature 425: 686–691. doi: 10.1038/nature02026 14562095 10.1038/nature02026

[30] Kornberg A, Pricer WE (1950) NUCLEOTIDE PYROPHOSPHATASE. Journal of Biological Chemistry 182: 763–778.

[31] Ogawa T, Yoshimura K, Miyake H, Ishikawa K, Ito D, et al (2008) Molecular Characterization of Organelle-Type Nudix Hydrolases in Arabidopsis. Plant Physiol 148: 1412–1424. doi: 10.1104/pp.108.128413 18815383 10.1104/pp.108.128413 PMC2577243

[32] Abdelraheim SR, Spiller DG, McLennan AG (2003) Mammalian NADH diphosphatases of the Nudix family: cloning and characterization of the human peroxisomal NUDT12 protein. Biochem J 374: 329–335. doi: 10.1042/BJ20030441 12790796 10.1042/BJ20030441 PMC1223609

[33] Goncalves AMD, Fioravanti E, Stelter M, McSweeney S (2009) Structure of an N-terminally truncated Nudix hydrolase DR2204 from Deinococcus radiodurans. Acta Crystallographica Section F 65: 1083–1087. 10.1107/S1744309109037191 PMC2777031 19923723

[34] Lee RS, Ford HC (1988) 5'-Nucleotidase of human placental trophoblastic microvilli possesses cobalt-stimulated FAD pyrophosphatase activity. Journal of Biological Chemistry 263: 14878–14883. 2844789

[35] Shin HJ, Mego JL (1988) A rat liver lysosomal membrane flavin-adenine dinucleotide phosphohydrolase: Purification and characterization. Archives of Biochemistry and Biophysics 267: 95–103. 2848456 10.1016/0003-9861(88)90012-4

[36] Tirrell I, Wall J, Daley C, Denial S, Tennis F, et al (2006) YZGD from Paenibacillus thiaminolyticus, a pyridoxal phosphatase of the HAD (haloacid dehalogenase) superfamily and a versatile member of the Nudix (nucleoside diphosphate x) hydrolase superfamily. doi: 10.1042/BJ20051172 16336194 10.1042/BJ20051172 PMC1383716

[37] Xu W, Gauss P, Shen J, Dunn CA, Bessman MJ (2002) The Gene e.1 (nudE.1) of T4 Bacteriophage Designates a New Member of the Nudix Hydrolase Superfamily Active on Flavin Adenine Dinucleotide, Adenosine 5′-Triphospho-5′-adenosine, and ADP-ribose. Journal of Biological Chemistry 277: 23181–23185. doi: 10.1074/jbc.M203325200 11976345 10.1074/jbc.M203325200

[38] McLennan A (2006) The Nudix hydrolase superfamily. Cellular and Molecular Life Sciences 63: 123–143. doi: 10.1007/s00018-005-5386-7 16378245 10.1007/s00018-005-5386-7 PMC11136074

[39] Cialabrini L, Ruggieri S, Kazanov MD, Sorci L, Mazzola F, et al (2013) Genomics-Guided Analysis of NAD Recycling Yields Functional Elucidation of COG1058 as a New Family of Pyrophosphatases. PLoS ONE 8: e65595 doi: 10.1371/journal.pone.0065595 23776507 10.1371/journal.pone.0065595 PMC3680494

[40] Bafunno V, Giancaspero TA, Brizio C, Bufano D, Passarella S, et al (2004) Riboflavin Uptake and FAD Synthesis in Saccharomyces cerevisiae Mitochondria. Journal of Biological Chemistry 279: 95–102. doi: 10.1074/jbc.M308230200 14555654 10.1074/jbc.M308230200

[41] Tzagoloff A, Jang J, Glerum DM, Wu M (1996) FLX1 Codes for a Carrier Protein Involved in Maintaining a Proper Balance of Flavin Nucleotides in Yeast Mitochondria. Journal of Biological Chemistry 271: 7392–7397. 8631763 10.1074/jbc.271.13.7392

[42] Costanzo M, Baryshnikova A, Bellay J, Kim Y, Spear ED, et al (2010) The genetic landscape of a cell. Science 327: 425–431. doi: 10.1126/science.1180823 20093466 10.1126/science.1180823 PMC5600254

[43] Brizio C, Galluccio M, Wait R, Torchetti EM, Bafunno V, et al (2006) Over-expression in Escherichia coli and characterization of two recombinant isoforms of human FAD synthetase. Biochemical and Biophysical Research Communications 344: 1008–1016. doi: 10.1016/j.bbrc.2006.04.003 16643857 10.1016/j.bbrc.2006.04.003

[44] Galluccio M, Brizio C, Torchetti EM, Ferranti P, Gianazza E, et al (2007) Over-expression in Escherichia coli, purification and characterization of isoform 2 of human FAD synthetase. Protein Expression and Purification 52: 175–181. doi: 10.1016/j.pep.2006.09.002 17049878 10.1016/j.pep.2006.09.002

[45] Ruiz A, Hurtado C, Ribeiro JM, Sillero A, Sillero MG (1989) Hydrolysis of bis (5'-nucleosidyl) polyphosphates by Escherichia coli 5'-nucleotidase. Journal of bacteriology 171: 6703–6709. 2556371 10.1128/jb.171.12.6703-6709.1989 PMC210566

[46] Nakane S, Wakamatsu T, Masui R, Kuramitsu S, Fukui K (2011) In vivo, in vitro, and X-ray crystallographic analyses suggest the involvement of an uncharacterized TIM barrel protein in protection against oxidative stress. Journal of Biological Chemistry: jbc. M111. 293886. 10.1074/jbc.M111.293886 PMC3308873 21984829

[47] Walker GM (2004) Metals in Yeast Fermentation Processes Advances in Applied Microbiology: Academic Press pp. 197–229. doi: 10.1016/S0065-2164(04)54008-X 10.1016/S0065-2164(04)54008-X 15251282

[48] Navarrete C, Petrezsélyová S, Barreto L, Martínez JL, Zahrádka J, et al (2010) Lack of main K+ uptake systems in Saccharomyces cerevisiae cells affects yeast performance in both potassium-sufficient and potassium-limiting conditions. FEMS Yeast Research 10: 508–517. doi: 10.1111/j.1567-1364.2010.00630.x 20491939 10.1111/j.1567-1364.2010.00630.x

[49] Pinto RMa, Fraiz FJ, Cabezas A, Ávalos Mn, Canales J, et al (1999) Preparation of Riboflavin 4′,5′-Cyclic Phosphate by Incubation of Flavin-adenine Dinucleotide with Mn2+in the Absence of Riboflavin 5′-Phosphate Cyclase. Analytical Biochemistry 268: 409–411. 10075835 10.1006/abio.1998.3063

[50] Pourbaix M (1966) Atlas of Electrochemical Equilibria in Aqueous Solutions New York: Pergamon.

[51] Torchetti EM, Bonomi F, Galluccio M, Gianazza E, Giancaspero TA, et al (2011) Human FAD synthase (isoform 2): a component of the machinery that delivers FAD to apo-flavoproteins. FEBS Journal 278: 4434–4449. doi: 10.1111/j.1742-4658.2011.08368.x 21951714 10.1111/j.1742-4658.2011.08368.x

[52] Grossman E, Medalia O, Zwerger M (2012) Functional architecture of the nuclear pore complex. Annu Rev Biophys 41: 557–584. doi: 10.1146/annurev-biophys-050511-102328 22577827 10.1146/annurev-biophys-050511-102328

[53] Bakker BM, Bro C, Kötter P, Luttik MA, Van Dijken JP, et al (2000) The mitochondrial alcohol dehydrogenase Adh3p is involved in a redox shuttle in Saccharomyces cerevisiae. Journal of bacteriology 182: 4730–4737. 10940011 10.1128/jb.182.17.4730-4737.2000 PMC111347

[54] Marres CAM, de Vries S, Grivell LA (1991) Isolation and inactivation of the nuclear gene encoding the rotenone-insensitive internal NADH: ubiquinone oxidoreductase of mitochondria from Saccharomyces cerevisiae. European Journal of Biochemistry 195: 857–862. 1900238 10.1111/j.1432-1033.1991.tb15775.x

[55] Larsson C, Påhlman IL, Ansell R, Rigoulet M, Adler L, et al (1998) The importance of the glycerol 3-phosphate shuttle during aerobic growth of Saccharomyces cerevisiae. Yeast 14: 347–357. doi: 10.1002/(SICI)1097-0061(19980315)14:4<347::AID-YEA226>3.0.CO;2-9 9559543 10.1002/(SICI)1097-0061(19980315)14:4<347::AID-YEA226>3.0.CO;2-9

[56] Ren J, Wen L, Gao X, Jin C, Xue Y, et al (2009) DOG 1.0: illustrator of protein domain structures. Cell Res 19: 271–273. doi: 10.1038/cr.2009.6 19153597 10.1038/cr.2009.6

[57] Aliverti A, Curti B, Vanoni MA (1999) Identifying and Quantitating FAD and FMN in Simple and in Iron-Sulfur-Containing Flavoproteins Flavoprotein Protocols In: Chapman SK, Reid GA, editors: Humana Press pp. 9–23. 10.1385/1-59259-266-X:9 10494539

[58] Rzewuski G, Cornell KA, Rooney L, Bürstenbinder K, Wirtz M, et al (2007) OsMTN encodes a 5′-methylthioadenosine nucleosidase that is up-regulated during submergence-induced ethylene synthesis in rice (Oryza sativa L.). Journal of Experimental Botany 58: 1505–1514. doi: 10.1093/jxb/erm014 17339651 10.1093/jxb/erm014

[59] Amberg DC, Burke DJ, Strathern JN (2006) Random Spore Analysis in Yeast Cold Spring Harbor Protocols 2006: pdb.prot4162. 10.1101/pdb.prot4162 22485563

[60] Harju S, Fedosyuk H, Peterson KR (2004) Rapid isolation of yeast genomic DNA: Bust n' Grab. BMC Biotechnol 4: 8 doi: 10.1186/1472-6750-4-8 15102338 10.1186/1472-6750-4-8 PMC406510

[61] Goldstein AL, McCusker JH (1999) Three new dominant drug resistance cassettes for gene disruption in Saccharomyces cerevisiae. Yeast 15: 1541–1553. doi: 10.1002/(SICI)1097-0061(199910)15:14<1541::AID-YEA476>3.0.CO;2-K 10514571 10.1002/(SICI)1097-0061(199910)15:14<1541::AID-YEA476>3.0.CO;2-K

[62] Gardner JM, Jaspersen SL (2014) Manipulating the yeast genome: deletion, mutation, and tagging by PCR. Yeast Genetics: Methods and Protocols: 45–78. 10.1007/978-1-4939-1363-3_5 25213239

[63] Schneider CA, Rasband WS, Eliceiri KW (2012) NIH Image to ImageJ: 25 years of image analysis. Nat Meth 9: 671–675. 10.1038/nmeth.2089 PMC5554542 22930834

[64] Meisinger C, Pfanner N, Truscott K (2006) Isolation of Yeast Mitochondria In: Xiao W, editor. Yeast Protocol: Humana Press pp. 33–39. 10.1385/1-59259-958-3:033 16118422

[65] King TE (1967) [58] Preparation of succinate dehydrogenase and reconstitution of succinate oxidase In: Ronald W. Estabrook MEP, editor. Methods in Enzymology: Academic Press pp. 322–331.

[66] Kearney EB, Singer TP (1956) STUDIES ON SUCCINIC DEHYDROGENASE: I. PREPARATION AND ASSAY OF THE SOLUBLE DEHYDROGENASE. Journal of Biological Chemistry 219: 963–975. 13319317

[67] Reed LJ, Willms CR (1966) [50] Purification and resolution of the pyruvate dehydrogenase complex (*Escherichia coli*). Methods in enzymology 9: 247–265.

[68] Postma E, Verduyn C, Scheffers WA, Van Dijken JP (1989) Enzymic analysis of the crabtree effect in glucose-limited chemostat cultures of Saccharomyces cerevisiae. Applied and environmental microbiology 55: 468–477. 2566299 10.1128/aem.55.2.468-477.1989 PMC184133

[69] Gliszczyńska-Świgło A, Koziołowa A (2000) Chromatographic determination of riboflavin and its derivatives in food. Journal of Chromatography A 881: 285–297. 10905712 10.1016/s0021-9673(00)00200-4

[70] Sporty JL, Kabir M, Turteltaub KW, Ognibene T, Lin SJ, et al (2008) Single sample extraction protocol for the quantification of NAD and NADH redox states in Saccharomyces cerevisiae. Journal of separation science 31: 3202–3211. doi: 10.1002/jssc.200800238 18763242 10.1002/jssc.200800238 PMC2640230

[71] Tarassov K, Messier V, Landry CR, Radinovic S, Molina MMS, et al (2008) An in Vivo Map of the Yeast Protein Interactome. Science 320: 1465–1470. doi: 10.1126/science.1153878 18467557 10.1126/science.1153878

[72] Singh J, Tyers M (2009) A Rab escort protein integrates the secretion system with TOR signaling and ribosome biogenesis. Genes & Development 23: 1944–1958. 19684114 10.1101/gad.1804409 PMC2725937

[73] Teste MA, Duquenne M, Francois JM, Parrou JL (2009) Validation of reference genes for quantitative expression analysis by real-time RT-PCR in Saccharomyces cerevisiae. BMC Mol Biol 10: 99 doi: 10.1186/1471-2199-10-99 19874630 10.1186/1471-2199-10-99 PMC2776018

[74] Livak KJ, Schmittgen TD (2001) Analysis of Relative Gene Expression Data Using Real-Time Quantitative PCR and the 2−ΔΔCT Method. Methods 25: 402–408. doi: 10.1006/meth.2001.1262 11846609 10.1006/meth.2001.1262

