# Supplementary figures and images for: Characterization of a non-nudix pyrophosphatase points to interplay between flavin and NAD(H) homeostasis in Saccharomyces cerevisiae
Source: PLoS One. 2018 Jun 14;13(6):e0198787. doi: 10.1371/journal.pone.0198787 (PMC6002036; doi:10.1371/journal.pone.0198787)

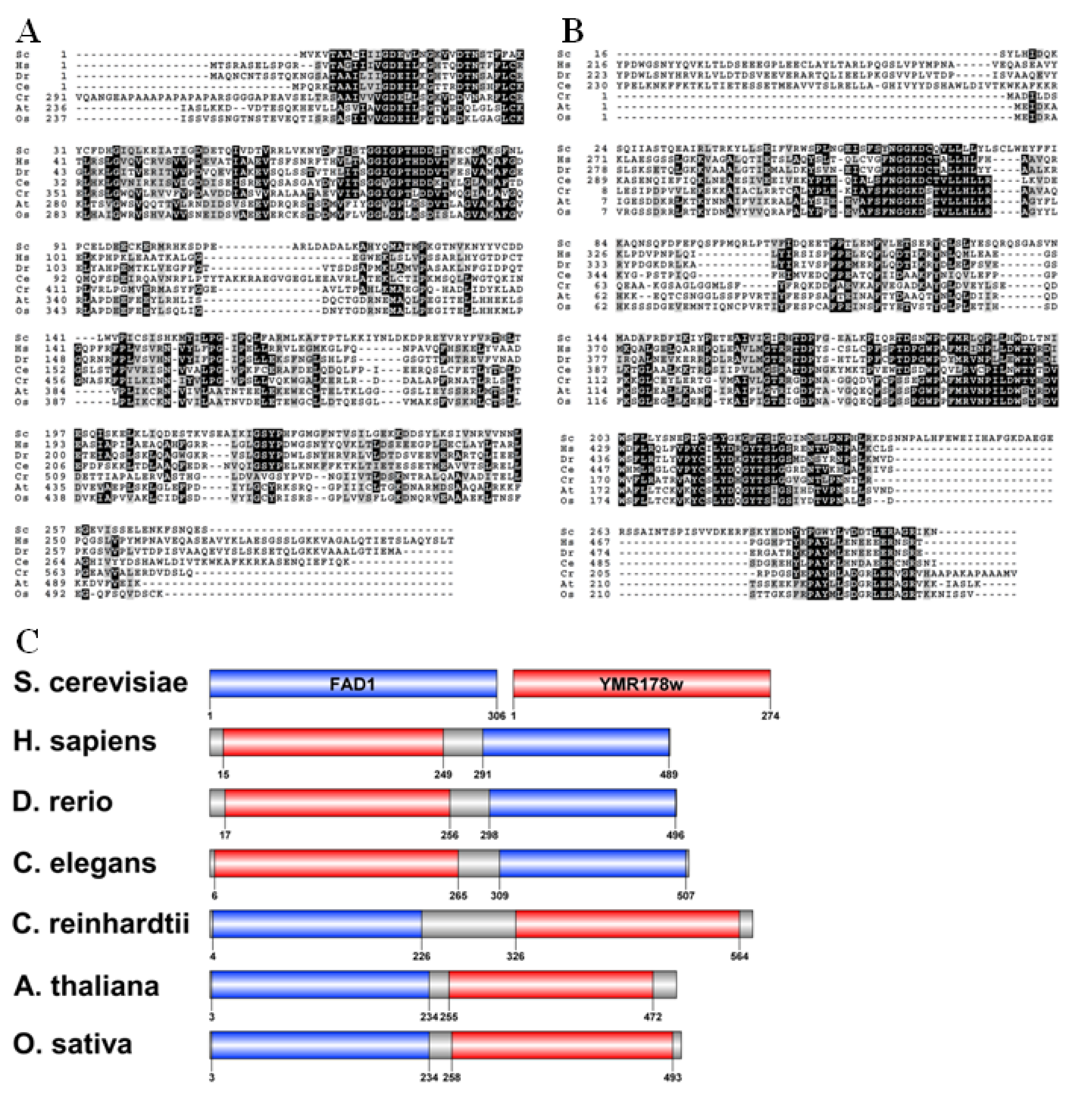

Supplement: S1 Fig — (A) Multiple sequence alignment of Fpy1p from S. cerevisiae with select proteins from other organisms. The species’ abbreviations are for Sc, Saccharomyces cerevisiae; Hs, Homo sapiens; Dr, Danio rerio; Ce, Caenorhabditis elegans; Cr, Chlamydomonas reinhardtii; At, Arabidopsis thaliana; and Os, Oryza sativa. (B) Multiple sequence alignment of Fad1p from S. cerevisiae with same proteins as in (A). (C) Illustrated domain structures of the proteins in A and B. Blue denotes domains with sequence homology to Fad1p, while Red denotes domains with sequence homology to Fpy1p. (TIF) [file pone.0198787.s001.tif]

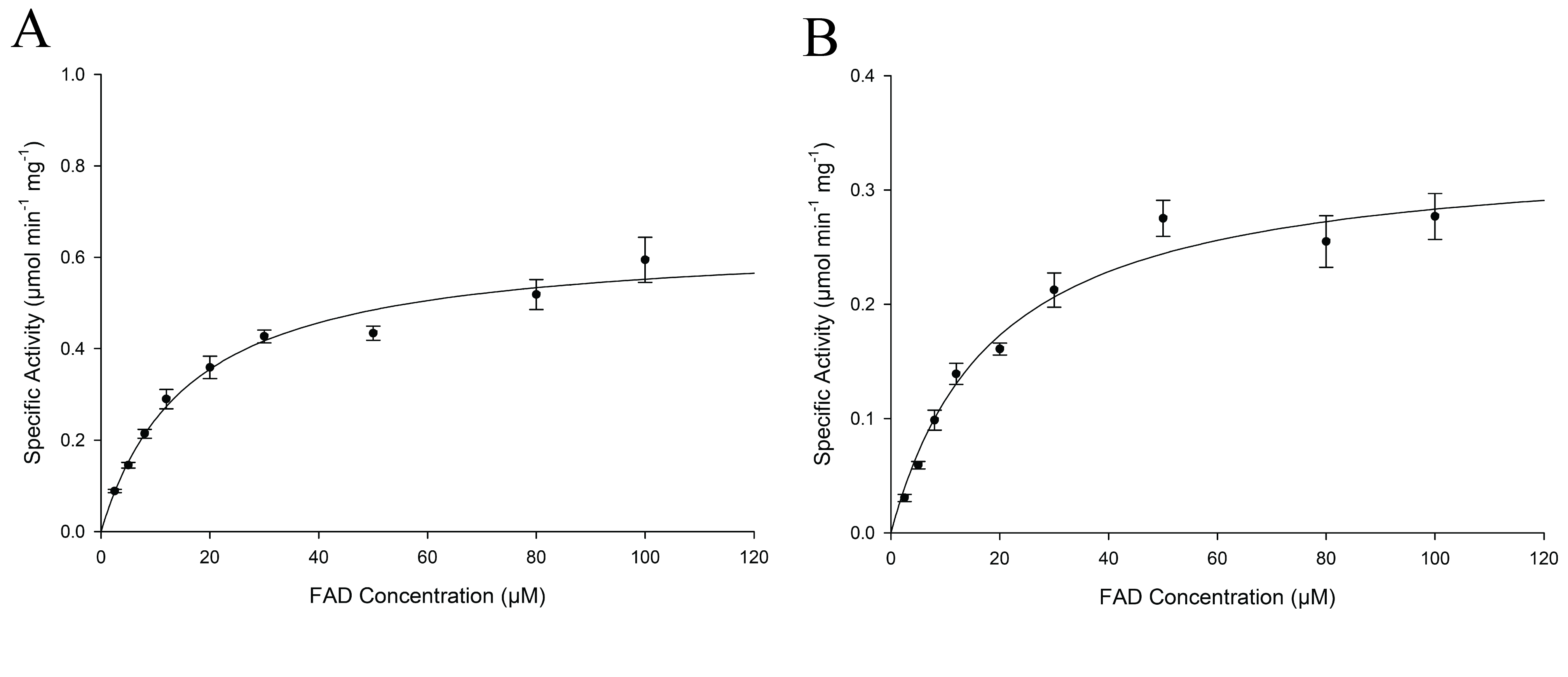

Supplement: S2 Fig — Measurements were made in the presence of either (A) 4 mM CoCl2 or (B) 10 mM MgCl2. Data is the average ± S.E. of three triplicate determinations. Curve is nonlinear fit to the Michaelis-Menten model using SigmaPlot 9.0. (TIF) [file pone.0198787.s002.tif]

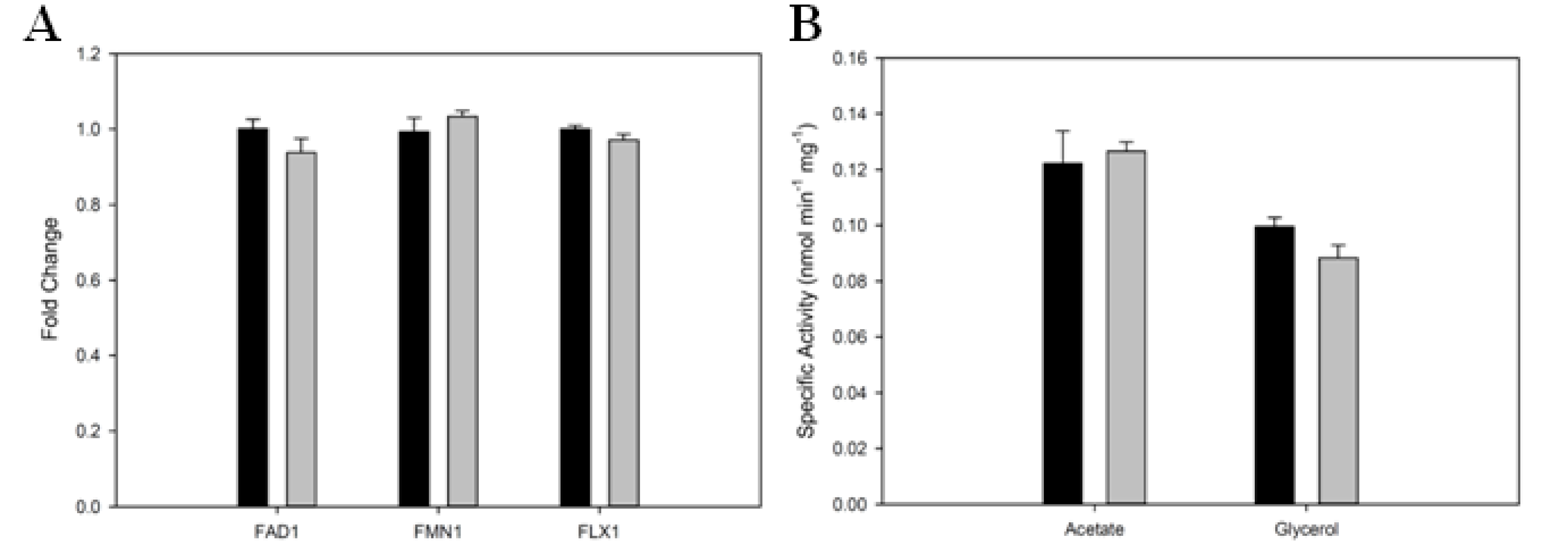

Supplement: S3 Fig — For both panels, solid black bar is wild-type control, solid grey bar is Δfpy1 deletion mutant. (A) Real-time PCR analysis of genes involved in flavin metabolism in yeast grown in YPD. Shown is the fold change relative to the housekeeping gene TFC1, calculated as described under “Experimental Procedures.” Data is the average ± S.E. of three technical replicates. (B) Mitochondrial FAD pyrophosphatase activity of yeast grown in YP supplemented with 1% sodium acetate or 3% glycerol. Assays were carried out as described under “Experimental Procedures” with 50 mM FAD and 10 mM MgCl2. Data is the average ± S.E. of three triplicate determinations. (TIF) [file pone.0198787.s003.tif]
