# Supplementary material for: Characterization of a non-nudix pyrophosphatase points to interplay between flavin and NAD(H) homeostasis in Saccharomyces cerevisiae
Source: PLoS One. 2018 Jun 14;13(6):e0198787. doi: 10.1371/journal.pone.0198787 (PMC6002036; doi:10.1371/journal.pone.0198787)
Supplement: S1 Table — (DOCX) [file pone.0198787.s004.docx]

| **S1 Table. Gradient for Separation of Derivatized AMP by HPLC.** A linear gradient between each step was used. | | | |
| --- | --- | --- | --- |
| Step | Time (min) | % Buffer A^a^ | % Buffer B^b^ |
| 1 | 0 | 100 | 0 |
| 2 | 1 | 90 | 10 |
| 3 | 2 | 60 | 40 |
| 4 | 3.4 | 90 | 10 |
| 5 | 5 | 90 | 10 |
| 6 | 7 | 100 | 0 |
| ^a^Buffer A is TBAS (5.7 mM tetrabutylammonium hydrogen sulfate, 30.5 mM KH_2_PO_4_, pH 5.8)  ^b^Buffer B is 1:2 TBAS:acetonitrile | | | |
