# Supplementary material for: Characterization of a non-nudix pyrophosphatase points to interplay between flavin and NAD(H) homeostasis in Saccharomyces cerevisiae
Source: PLoS One. 2018 Jun 14;13(6):e0198787. doi: 10.1371/journal.pone.0198787 (PMC6002036; doi:10.1371/journal.pone.0198787)
Supplement: S2 Table — (DOCX) [file pone.0198787.s005.docx]

| **S2 Table: Primers for PCR Verification of Gene Deletion.** Amplification with the genomic upstream and genomic gene-specific primer pair is possible only if the ORF is present. Amplification with the genomic upstream and deletion cassette specific primer pair is possible only if the ORF has been replaced by the deletion cassette. | | | |
| --- | --- | --- | --- |
| Target Gene | Genomic Upstream | Genomic Gene-Specific | Deletion Cassette Specific |
| FPY1 | CGAGGGATATAACAATAGCAGTCAT | TAGGTGTGTTCTGACAAAATAACGA | CTGCAGCGAGGAGCCGTAAT |
| FLX1 | CTTTTCAGTTTCCTTCCGTTTATTT | ACCCAGATAGGGTTTGTTAGGATAG | CTGCAGCGAGGAGCCGTAAT |
