# Supplementary material for: Characterization of a non-nudix pyrophosphatase points to interplay between flavin and NAD(H) homeostasis in Saccharomyces cerevisiae
Source: PLoS One. 2018 Jun 14;13(6):e0198787. doi: 10.1371/journal.pone.0198787 (PMC6002036; doi:10.1371/journal.pone.0198787)
Supplement: S3 Table — (DOCX) [file pone.0198787.s006.docx]

| **S3 Table: Primers for Real-Time PCR** | | |
| --- | --- | --- |
| Target Gene | Forward Primer | Reverse Primer |
| FAD1 | CCCATGCAAAGACTTCCAAC | ATGCGTCTGCCATATTGACC |
| FMN1 | TCCGCCGAATTGGGTATT | CTTCCATCCCGCCTTGTTT |
| FLX1 | TACAAGTAAAGGCGCCCAAG | TAAGGCACCTTGCGAAACAC |
| TFC1 | CCAGGCCCCACCGTTAATAG | ACTTTTTCGATGCCCCCACA |
| UBC6 | TGGACGTTTCAAGCCCAACA | TCGTGGCTTCATCACTGGTC |
| ALG9 | ATTGACATCGTCGCCCCAAT | GGTTGATTGGCTCCGGTACG |
